# Unusual Activity of Rationally Designed Cobalt Phosphide/Oxide Heterostructure Composite for Hydrogen Production in Alkaline Medium

**DOI:** 10.1021/acsnano.1c09254

**Published:** 2022-03-07

**Authors:** Merfat M. Alsabban, Mathan Kumar Eswaran, Karthik Peramaiah, Wandi Wahyudi, Xiulin Yang, Vinoth Ramalingam, Mohamed. N. Hedhili, Xiaohe Miao, Udo Schwingenschlögl, Lain-Jong Li, Vincent Tung, Kuo-Wei Huang

**Affiliations:** † Division of Physical Sciences and Engineering, King Abdullah University of Science and Technology, Thuwal 23955-6900, Kingdom of Saudi Arabia; ‡ KAUST Catalysis Center, King Abdullah University of Science and Technology, Thuwal 23955-6900, Kingdom of Saudi Arabia; § Department of Chemistry, University of Jeddah, Jeddah 21959, Kingdom of Saudi Arabia; ∥ Core Laboratories, King Abdullah University of Science and Technology, Thuwal 23955-6900, Kingdom of Saudi Arabia; ⊥ Department of Mechanical Engineering, The University of Hong Kong, Pokfulam Road, Hong Kong

**Keywords:** cobalt phosphide, cobalt mixed oxides, electrochemical catalyst, hydrogen evolution reaction (HER), phosphatization

## Abstract

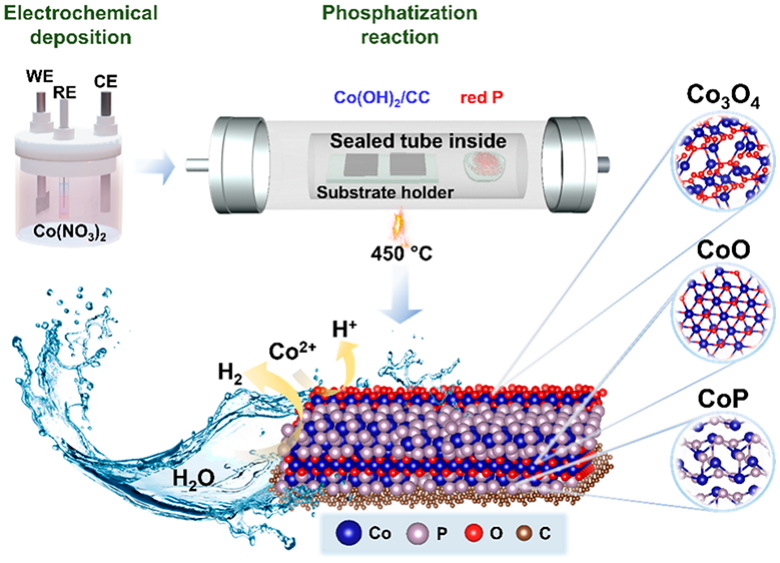

Design and development of an efficient, nonprecious catalyst with structural features and functionality necessary for driving the hydrogen evolution reaction (HER) in an alkaline medium remain a formidable challenge. At the root of the functional limitation is the inability to tune the active catalytic sites while overcoming the poor reaction kinetics observed under basic conditions. Herein, we report a facile approach to enable the selective design of an electrochemically efficient cobalt phosphide oxide composite catalyst on carbon cloth (CoP-Co_*x*_O_*y*_/CC), with good activity and durability toward HER in alkaline medium (η_10_ = −43 mV). Theoretical studies revealed that the redistribution of electrons at laterally dispersed Co phosphide/oxide interfaces gives rise to a synergistic effect in the heterostructured composite, by which various Co oxide phases initiate the dissociation of the alkaline water molecule. Meanwhile, the highly active CoP further facilitates the adsorption–desorption process of water electrolysis, leading to extremely high HER activity.

Storing low-carbon energy in the form of molecular hydrogen (H_2_) as a clean energy carrier, holds tantalizing prospects to reshape the current energy consumption away from fossil fuels.^1,2^ Electrocatalytic water splitting has been researched as one of the most efficient technologies to produce hydrogen as it provides an opportunity to directly integrate with sustainable renewable energy resources such as wind and solar power, and so on.^3,4^ In this regard, electrocatalytic hydrogen production under alkaline electrolytes has been identified as a viable industrial technology to generate large-scale high-purity hydrogen fuels.^5^ However, slow cathodic HER kinetics in alkaline solution leads to a low hydrogen production efficiency.^6^ Thus, far, precious Pt and Pt-based materials have been widely utilized as state-of-the-art catalysts for driving HER.^7^ However, these noble metals have significantly suffered from problems toward industrial-scale hydrogen production due to their high costs, scarcity, and poor durability.^8,9^ To address these issues, it is imperative to rationally develop an efficient nonprecious metal catalyst with superior HER activity and stability under alkaline electrolytes.

The deployment of nonprecious transition metals (TMs), such as Co, Ni, Fe, Mo, and W, has demonstrated tremendous promises for catalyzing efficient HER and oxygen evolution reaction (OER).^10−19^ In particular, cobalt phosphides (CoP) have been realized as a promising HER catalyst owing to its low cost, efficient catalytic activity, and long-term durability under both acidic and alkaline medium.^20,21^ Nevertheless, the HER activity of CoP-based catalysts needs to be further improved to achieve comparable hydrogen formation efficiency to noble metal catalysts. The HER activity of CoP has been enhanced via various strategies, including doping second transition metals, surface modification, defect engineering, and integration of the supporting material.^5,21,22^ For instance, Pan et al. studied a series of CoP-based electrocatalysts with different phase structures and supporting material, by which CoP/NCNTs catalyst achieved an overpotential of 99 mV to reach 20 mA cm^–2^ current density in an acidic medium.^11^ Likewise, Yuan and co-workers designed a CoP mesoporous nanorod array electrocatalyst using electrodeposition method. An overpotential of 54 mV was required to attain a current density 10 mA cm^–2^ in 1 M KOH_(aq)_ electrolyte solution.^23^ Very recently, Men et al., has modified the electronic structure on CoP via doping a series of second transition metals (Fe, Ni, Cu, Mo, Mn, V, and Cr).^5^ The Cr-CoP electrocatalyst exhibited an excellent HER performance in 1 M KOH_(aq)_ electrolyte with an overpotential of 36 mV at a current density of 10 mA cm^–2^. The reduced graphene oxide supported CoP electrocatalyst was reported as a bifunctional catalyst for HER and OER in 1 M KOH_(aq)_, delivering an HER overpotential of 134 mV at 10 mA cm^–2^.^22^ Nevertheless, in spite of these recent advancements, rational design of multiphases in CoP heterostructured electrocatalyst toward HER has been rarely investigated.

To boost catalytic activity, electrochemical stability, and utilization efficiency, an ideal HER catalyst should have (a) optimized geometric factors for high electrochemically active surface area (EASA) essential for overpotentials (η_10_), Tafel slopes, exchange current density, and efficient charge-transfer across electrolyte/electrode interfaces; (b) an HER-favorable chemical environment for intrinsic activity, such as turnover frequency (TOF), and an efficient electronic connection between active sites and current extracting substrates; and (c) a mechanism to maintain above-mentioned metrics for high tolerance at extreme pH values, for long periods of operation and at elevated temperatures. Besides, before truly appreciating the widespread adoption, the manufacturing process should be facile and scalable by eliminating assembly steps and bypassing complex postengineering processes to generate minimal waste. Herein, we overcome the mechanistic hurdles to catalyze efficient hydrogen production in alkaline medium by introducing multiphases Co–P–O heterostructured composites. Robust yields of different nanostructured phases of cobalt phosphides were consistently obtained. Electrochemical analysis indicated that CoP-Co_*x*_O_*y*_/CC (450 °C) heterostructured composite is the most active phase toward hydrogen formation, to which a current density of 10 mA cm^–2^ can be delivered at a low overpotential of only −43 mV in 1 M KOH_(aq)_. Density functional theory (DFT) calculations were also applied to reveal the motives behind the HER activity in alkaline water. It was found that the readily available Co^2+^ cations in alkaline water contribute to the formation of Co(OH)_2_, which stimulates water dissociation, while adjacent Co sites in CoP promote the adsorption of hydrogen intermediates followed by their recombination into hydrogen molecules. This work provides (i) insights into the phase transition of CoP system^24^ and (ii) understanding of the heterostructured catalytic function for enhancing the kinetics of hydrogen production in an alkaline medium.

## Results and Discussion

### Phase Transition of the CoP System upon Annealing and Phosphatization

**Figure 1:**
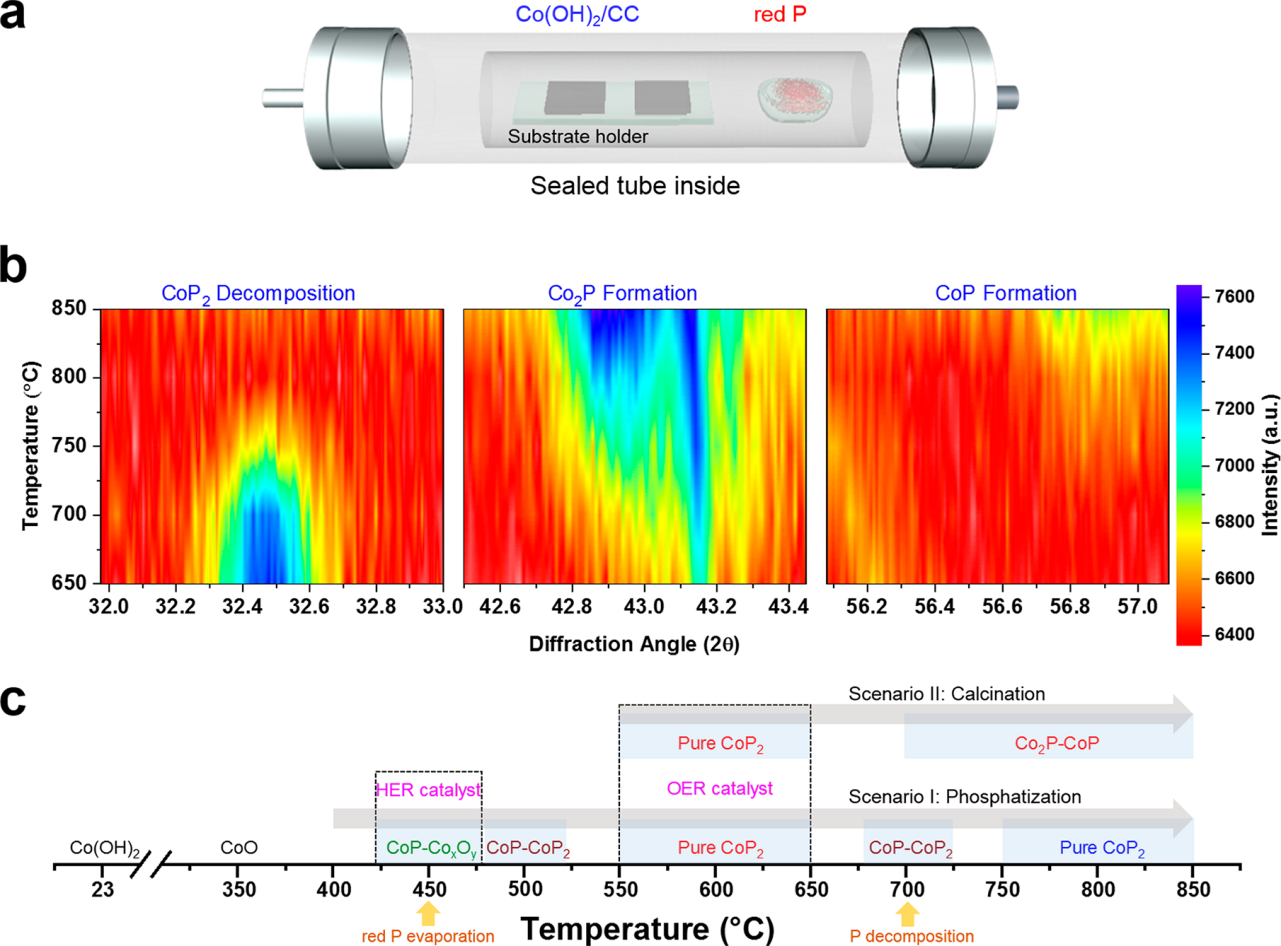
(a) Schematic representation of the phosphatization process. (b and c) CoP_*x*_ evolution as a function of the phosphatization temperature and XRD angle, revealing CoP_2_ decomposition and formation of Co_2_P and CoP as the temperature elevates.

The CVD strategy used in this study provides systematic control of Co and P stoichiometric ratio, which could afford a successive formation of nanostructured CoP_*x*_/CC with spatially interspersed phases. Figure 1a demonstrates the schematic presentation of the setup of different phases of CoP_*x*_/CC through facile two-steps CVD approach. First, Co-oxy species were electrodeposited on CC substrate, followed by gas-phase phosphatization at selected temperatures (450–850 °C) as described in our previous report.^12^ X-ray diffraction (XRD) patterns of all the obtained phases were analyzed (Figure S2), with detailed phase composition and crystal structure in Tables S1 and S2. Distinctive peaks at 2θ = 25.8, 43.5, and 52.3° correspond to CC substrate.^25^ When the starting material (Co(OH)_2_/CC) was phosphatized to 450 °C, partial phosphatization occurred, and a mixture of CoP/CC, CoO/CC, and Co_3_O_4_/CC is obtained. However, raising the temperature to 550 and 650 °C induced the formation of monoclinic CoP_2_/CC without any other detectable phases. The progressive phase transition upon the phosphatization process is ascribed to complete evaporation of red P at ∼550 °C, as confirmed by thermogravimetric analysis (TGA) in Figure S3a. Further phosphatization at higher temperatures ranging from 750–850 °C led to the reformation of highly crystalline orthorhombic CoP/CC. At metaphases temperature of 500 °C, the multiphases composition of CoP/CC and CoP_2_/CC is detected in alignment with those formed at 700 °C but with higher crystallinity signified by sharper peaks. Moreover, we have found that the phase transition from CoP_2_/CC (550–650 °C) to CoP/CC (750–850 °C) may be associated with CoP_2_/CC decomposition at elevated temperatures. To further confirm the P-induced phase transition, the as-prepared CoP_2_/CC (650 °C) specimen was annealed (in the absence of red P) and phosphatized (in the presence of red P) to 850 °C. XRD analysis (Figure S3b) shows that annealing of CoP_2_/CC (650 °C) sample to 850 °C led to an asymmetric distribution of stoichiometry with minor CoP/CC content, while Co_2_P/CC is overwhelmingly abundant. The discrepancy in stoichiometric distribution therefore attests to our assumption where decomposition of CoP_2_/CC at high temperatures is the main driving force of phase transformation. In contrast, the phosphatization process resulted in a complete conversion of CoP_2_/CC (650 °C) to CoP/CC nanostructures with high crystallinity. Indeed, these results clarify the phenomena of phase evolution-degradation of CoP-based materials in terms of increasing temperature as follows: (i) Initial phase formation (CoP-Co_*x*_O_*y*_/CC) occurred at 450 °C as a result of partial phosphatization of Co(OH)_2_/CC starting precursor due to incomplete degradation of red P. (ii) Pure CoP_2_/CC began to form at 550 °C, which can be ascribed to complete evaporation of P at a particular temperature as identified in TG profile. (iii) As the phosphatization temperatures increase (>720 °C), CoP_2_/CC started to decompose along with volatilization of P. Reproduction of pure CoP/CC takes place, whereas temperature elevation of annealing process results in the formation of a mixture of CoP and Co_2_P.

Later, the mechanistic insights into the phase transition of CoP_2_/CC to form CoP/CC and Co_2_P/CC phases were studied. We carried out an *in situ* temperature-dependent XRD analysis for the CoP_2_/CC (650 °C) specimen at annealing temperature from 650 to 850 °C. The results presented in the 2D XRD spectra (Figure 1b) reveal that the annealing process led to an apparent attenuation of CoP_2_/CC (2θ = 32.46°) and gradual growth of Co_2_P/CC (2θ = 43.3°) and CoP/CC (2θ = 56.9°) phases (see Figure S4 for details). Figure 1c summarizes the phase transition of CoP_*x*_/CC upon the annealing and phosphatization process obtained from our results.

Scanning transmission electron microscopy (STEM) analysis shows that CoP/CC particles exhibited an irregular structure, while Co_2_P/CC particles formed a regular morphology and well-developed crystal facets (Figure S5a). Besides, accompanied STEM-EDX elemental composition analysis confirms the formation of CoP/CC and Co_2_P/CC phases, with no detection of oxygen contamination. Further, high-resolution transmission electron microscopy (HR-TEM) imaging in Figure S5b displays *d*-spacing and lattice symmetry of 3.3 Å for (200) and 2.2 Å for (221), which are consistent with the orthorhombic phase of Co_2_P/CC. Microstructure analysis of all the phases was accomplished using field-emission scanning electron microscopy (FESEM). Turning into the samples obtained from the phosphatization process, Figure S6 shows individual nanosized particles that appear on top of material film at 500 °C and then start to sinter at higher temperatures up to 850 °C, which is a typical process for thermal treatments. However, surface analysis of the samples by high-resolution X-ray photoelectron spectroscopy (XPS) in Figures S7 and S8 show a Co/P ratio of 1.00 and 0.34 for those phosphatized at 850 and 650 °C, in which the values are comparable to the theoretical Co/P ratio of CoP and CoP_2_, respectively.^24^ The results further confirm the significance of phosphatization to control the phase transition of CoP_*x*_ upon the thermal treatment.

The key roles of different phases obtained from the phosphatization process are potentially helpful for electrocatalytic reactions in different environments. We have previously reported that CoP_2_/CC obtained after phosphatization to 650 °C shows the most active phase toward OER in alkaline solutions.^12^ In the present work, CoP-Co_*x*_O_*y*_/CC formed at 450 °C shows an unusual activity of the HER in base compared to the subsequent phases formed at higher phosphatization temperatures. The detailed properties of heterostructured CoP-Co_*x*_O_*y*_/CC composite and their relations with the HER performance are studied further in the following section.

### Catalyst Characterizations

**Figure 2:**
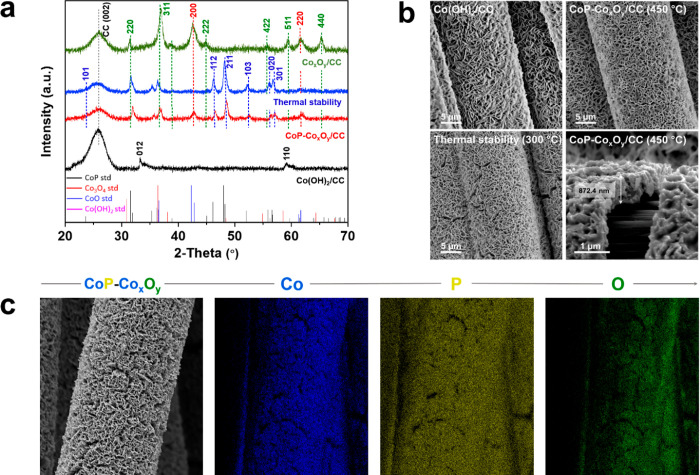
(a) XRD patterns of Co(OH)_2_/CC and CoP-Co_*x*_O_*y*_/CC formed after phosphatization at 450 °C, thermal stability after annealing at 300 °C, and Co_*x*_O_*y*_/CC formed after annealing at 450 °C. (b) SEM images of electrodeposited Co(OH)_2_/CC and CoP-Co_*x*_O_*y*_/CC (450 °C), thermal stability (300 °C), and cross-sectional SEM image of CoP-Co_*x*_O_*y*_/CC (450 °C). (c) SEM image and corresponding EDX elemental mapping of Co, P, and O for CoP-Co_*x*_O_*y*_/CC (450 °C).

Phase identity and crystalline structure of the catalyst were initially examined by XRD analysis. Figure 2a demonstrates the XRD pattern of electrodeposited Co-oxy species on CC supporting substrate, which is primarily indexed to Co(OH)_2_/CC (black curve). As previously stated, partial phosphatization occurs when the catalyst is phosphatized to 450 °C, and a mixture of CoP, CoO, and Co_3_O_4_ are detected (red curve). In addition, the thermally robust (300 °C) specimen shows identical diffraction peaks and, consequently, confirming similar composition (blue curve). For comparison, annealing of the Co(OH)_2_/CC precursor to 450 °C in the absence of red P is ascribed to CoO and Co_3_O_4_ (green curve). Figure 2b compares the surface configuration of the Co(OH)_2_/CC precursor, CoP-Co_*x*_O_*y*_/CC (450 °C), along with its thermal stability carried at 300 °C. Full coverage of sturdily attached rugged-like assembly of Co(OH)_2_ on CC fibers is observed,^17^ which remains intact after being phosphatized to 450 °C and even after being subjected to thermal stability test at 300 °C. The SEM cross-sectional image of entirely exfoliated CoP-Co_*x*_O_*y*_ film from CC single fiber is examined, and catalyst thickness was approximated to be ∼870 nm. The SEM image of a single carbon fiber coated with as-prepared composite is shown in Figure 2c. The corresponding energy-dispersive X-ray (EDX) elemental mapping of Co (blue), P (yellow), and O (green) further confirm the homogeneous distribution of constituent elemental components on the CC substrate.

**Figure 3:**
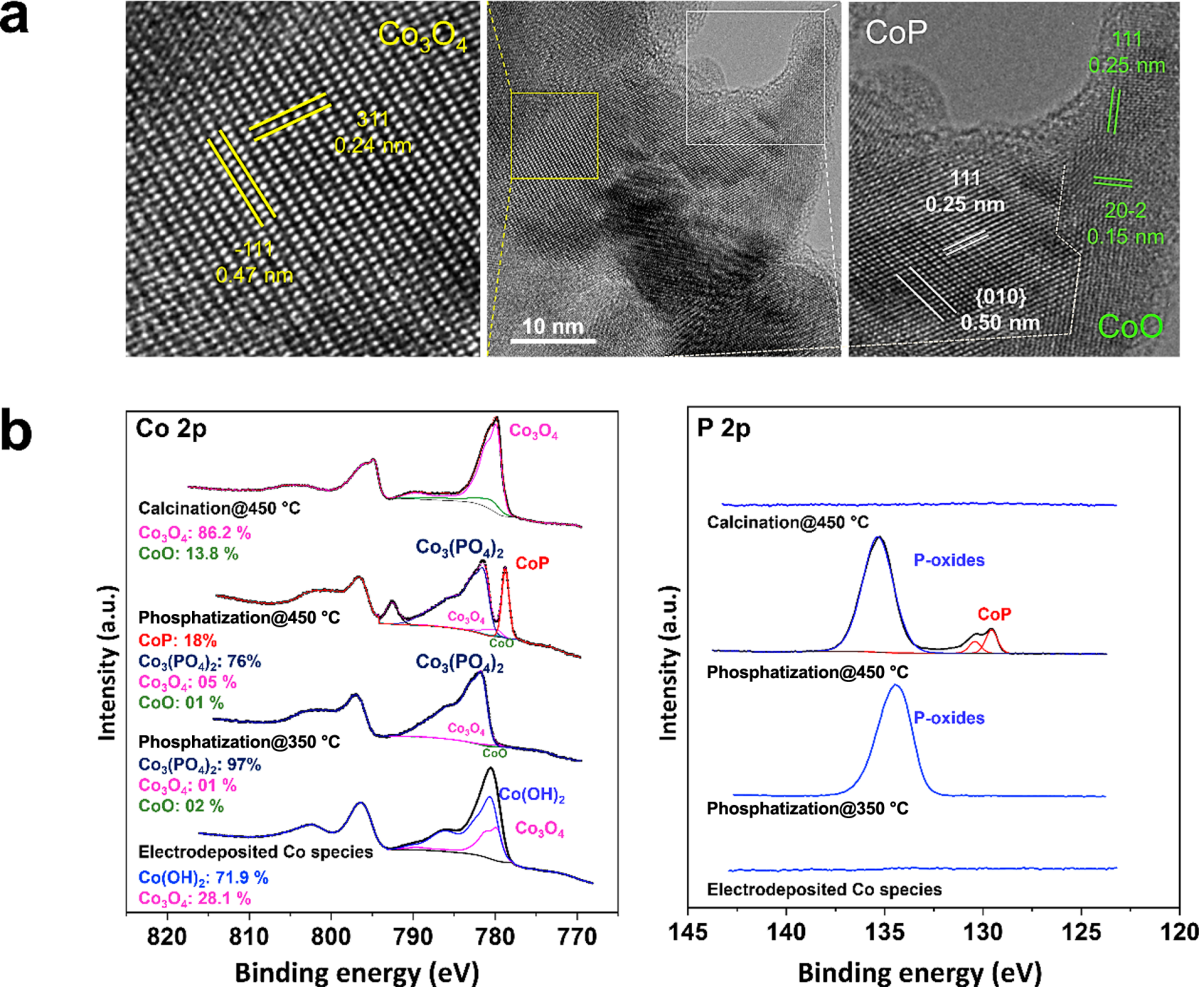
(a) HR-TEM image of CoP-Co_*x*_O_*y*_/CC formed at 450 °C. (b) Narrow scan Co 2p and P 2p XPS spectra of Co-oxy species/CC, the phosphatized specimen at 350 and 450 °C, and the calcined sample at 450 °C.

To further investigate the chemical composition, TEM analysis was carried out. Figure 3a shows a high-resolution transmission electron microscopic image (HR-TEM) of a sample formed at 450 °C. The HR-TEM images exhibit the typical (111) and (010) lattice sets of CoP with *d*-spacing of 0.25 and 0.50 nm, respectively. Moreover, it reveals a heterogeneous chemical composition in terms of CoO and Co_3_O_4_ phase distribution, as illustrated by their lattice planes.

Surface chemical states and composition of electrodeposited Co-oxy species on CC and samples synthesized after being phosphatized to 350 and 450 °C, in addition to annealed sample at 450 °C, were further studied using XPS analysis. The survey scan spectra for each sample shows the existence of Co, O, P, and C (Figure S9). For all samples, narrow scan XPS spectra for Co 2p and P 2p were obtained (Figure 3b). In the case of Co-oxy species/CC, the Co 2p core-level spectrum shows two main peaks located at 780.6 and 796.5 eV attributed to a Co 2p_3/2_ and Co 2p_1/2_ doublet accompanied by their satellites at 786.3 and 802.3 eV, respectively. The different cobalt oxidation states have been identified through Co 2p_3/2_ peak fitting similar to Biesinger et al. approach.^26,27^ The Co 2p_3/2_ spectrum of the Co-oxy species/CC is well-fit with the following information from standard samples of Co(OH)_2_ and Co_3_O_4_.^26,28^ It is identified that Co(OH)_2_ is the dominant oxy-species of cobalt oxides with ∼72%, while Co_3_O_4_ forms ∼28% of the sample surface. The Co-oxy species/CC was then phosphatized to 350 °C. Two main peaks centered at 781.9 and 798.1 eV corresponding to a Co 2p_3/2_ and Co 2p_1/2_ doublet, along with their broad satellites around 786.4 and 803.7 eV, respectively, are observed in the Co 2p core-level spectrum. The position of the peaks is found to be at higher binding energy from all reported cobalt oxides.^26^ The shape of the spectrum and peaks positions are similar to those originated from cobalt phosphate; in particular, the Co_3_(PO_4_)_2_ species.^29,30^ Besides, P 2p core-level spectrum shows a single broad peak located at 134.4 eV ascribed to phosphorus from Co_3_(PO_4_)_2_.^29,30^

When the phosphatization of Co-oxy species/CC is carried out at 450 °C, two new peaks located at 778.8 and 793.8 eV are observed which correspond to Co 2p_3/2_ and Co 2p_1/2_ of CoP.^14,17,31^ Remaining peaks positioned at 781.7 and 798.1 eV, accompanied by their satellites around 786.5 and 803.1 eV are corresponding to dominant species of Co_3_(PO_4_)_2_;^29,30^ however, the contribution of Co-oxides is not excluded. This is further confirmed by the corresponding P 2p spectrum where the peaks again appeared at 129.6 and 130.4 eV that are attributed to P 2p doublet from CoP which are not observed at 350 °C,^14,17,31^ along with a broad peak observed around ∼135.2 eV assigned to phosphorus cations in highly oxidized states. For comparison, Co-oxy species/CC was annealed at 450 °C in the absence of elemental red P precursor; two prominent peaks are observed in Co 2p core-level spectrum at 779.7 and 794.8 eV attributed to Co 2p_3/2_ and Co 2p_1/2_ doublet related with their satellites around 789.7 and 804.8 eV, respectively. The Co 2p_3/2_ spectrum of Co_*x*_O_*y*_ (450 °C) is well fitted using the information on standard samples of CoO and Co_3_O_4_.^26,28^ Moreover, the Co_3_O_4_ form of oxides is found to be the most dominant oxy-species with ∼86%, whereas CoO forms only 14% of the sample surface. These observations demonstrate that the surface chemical composition of the sample calcined at 450 °C in the absence of P is different from the one being phosphatized at a similar temperature, and consequently, the different surface activity will take place during HER reaction.

### Electrocatalytic Activity for HER

**Figure 4:**
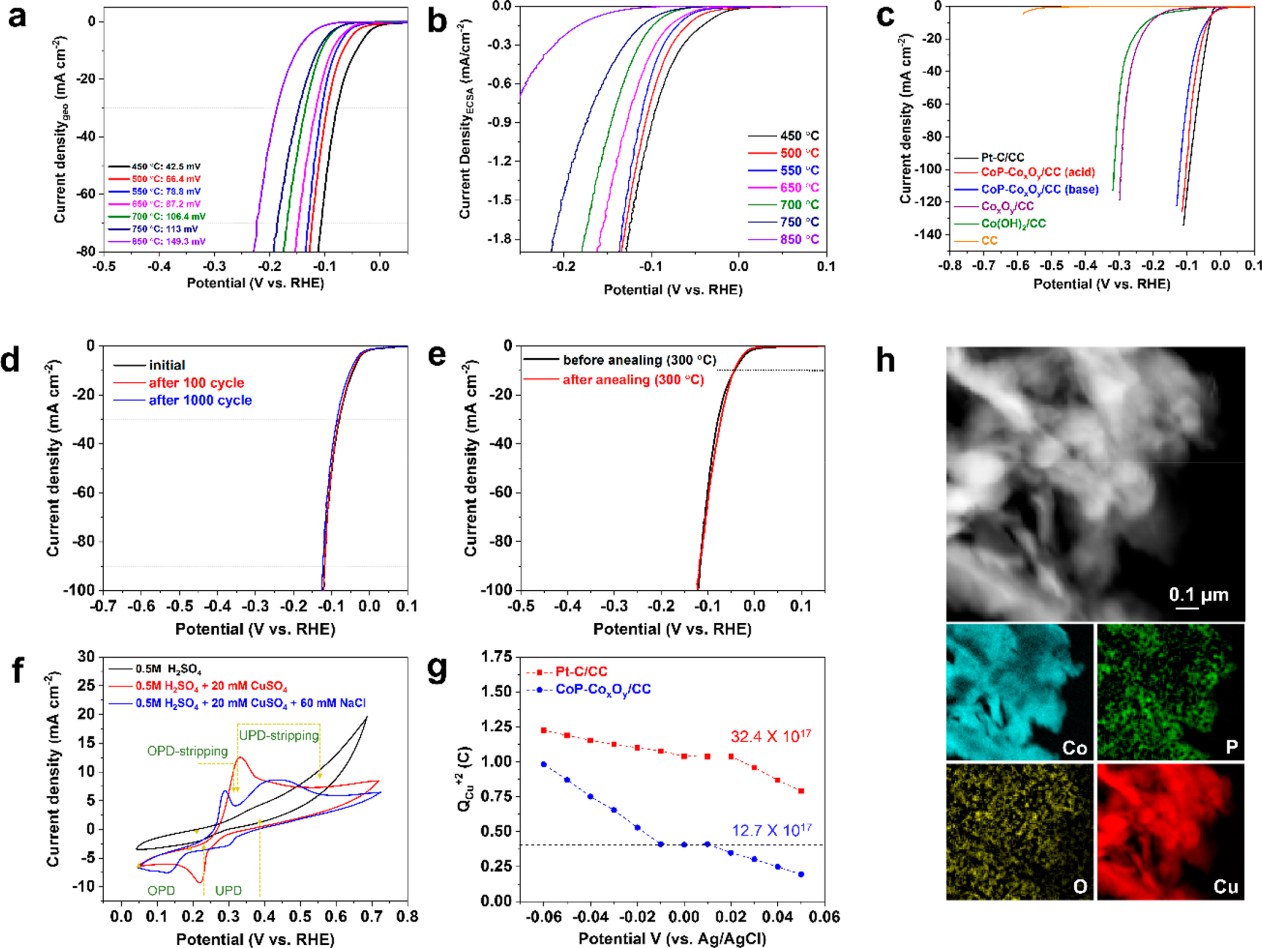
(a) Polarization curves at a scan rate of 0.1 mV s^–1^ in 1 M KOH_(aq)_ electrolyte solution for CoP-Co_*x*_O_*y*_/CC (450 °C) and CoP_*x*_/CC formed at different phosphatization temperatures. The current was normalized by the geometrical area of the carbon cloth substrate, and the potential was measured after internal resistance correction. (b) ECSA-normalized LSV curves carried out at 0.1 mV s^–1^ in 1 M KOH_(aq)_ electrolyte solution for CoP-Co_*x*_O_*y*_/CC (450 °C) and CoP_*x*_/CC formed at different phosphatization temperatures. (c) Polarization curves of 20% Pt–C/CC, CoP-Co_*x*_O_*y*_/CC (450 °C), Co_*x*_O_*y*_/CC (450 °C), Co(OH)_2_/CC, and pristine CC at a scan rate of 0.1 mV s^–1^ in 1 M KOH_(aq)_ electrolyte solution. (d) Polarization curves of CoP-Co_*x*_O_*y*_/CC (450 °C) at a scan rate of 0.1 mV s^–1^ for durability testing. (e) Polarization curves of pristine CoP-Co_*x*_O_*y*_/CC (450 °C) and after annealing to 300 °C with a scan rate of 0.1 mV s^–1^ in 1 M KOH_(aq)_ electrolyte solution. (f) CV curves of CoP-Co_*x*_O_*y*_/CC scanned in different solutions. (g) Required charges to strip deposit Cu at different underpotentials (−0.06 to 0.05 V). (h) Co, P, O, and UPD Cu EELS mappings of CoP-Co_*x*_O_*y*_/CC.

The electrocatalytic HER performances of various phases formed at different phosphatization temperatures (450–850 °C) were evaluated via their polarization curves. The electrochemical reactions were conducted in a standard three-electrode configuration using 1 M KOH_(aq)_ electrolyte solution. The current densities were first normalized by its geometrical area of CC, and the potential was measured after internal resistance correction (Figure 4a), then re-evaluated in terms of its normalized electrochemical surface area, *J*_*ECSA*_ (electrolyte adsorption) (see Figure 4b). Uniquely, CoP-Co_*x*_O_*y*_/CC nanoparticles prepared during phosphatization at 450 °C have shown the lowest overpotential among the whole series. For comparison, different electrocatalysts were tested for their HER activities, including Pt–C/CC, Co_*x*_O_*y*_/CC (450 °C), Co(OH)_2_/CC, and pristine CC in 1 M KOH_(aq)_ (Figure 4c). By comparing the voltammetric response of these catalysts, it has been observed that Pt–C/CC yielded the highest hydrogen production activity with a close-to-zero overpotential as expected. In contrast, the pristine CC substrate shows no obvious effectiveness toward HER. The Co_*x*_O_*y*_/CC prepared through annealing process at 450 °C and the Co(OH)_2_/CC precursor exhibited an overpotential of 207 mV and 220 mV at 10 mA cm^–2^, respectively, whereas CoP-Co_*x*_O_*y*_/CC formed after phosphatization to 450 °C showed an outstanding HER activity with an extremely low overpotential of −43 mV in 1 M KOH_(aq)_ to reach the current density of 10 mA cm^–2^. This undoubtedly reveals the superior activity of CoP-Co_*x*_O_*y*_/CC composite nanoparticles to most lately reported CoP-based catalysts for hydrogen production in alkaline solutions (see Table S3 for details). This excellent HER performance is mainly attributed to the synergistic effect of both available Co^2+^ cations and adjacent Co-active sites in the dissociation of water and adsorption–desorption of hydrogen, respectively.

The HER electrochemical activity for a physical mixture of CoP, CoO, and Co_3_O_4_ was also examined to conclusively confirm the importance of heterointerfaces’ existence on HER enhancement. Prior investigation of chemical components of electrocatalyst is essential to prepare the physical mixture of the catalytic system. XRD data confirmed that CoP content is ∼76%, while both CoO and Co_3_O_4_ contents are only ∼19 and ∼5%, respectively. Accordingly, a physical mixture of CoP and Co_*x*_O_*y*_ was prepared and tested by combining two different electrodes (see Figure S10 for details). On the basis of the LSV curves, heterointerfaces presented in CoP and Co_*x*_O_*y*_ are crucial and play a vital role, in which CoP can tune the electronic properties of Co_*x*_O_*y*_ and enhances the overall HER activity under alkaline conditions.

To exclude any impact of the catalyst multicomposition, a pure CoP/CC sample was tested for its HER catalytic activity in the basic medium where the mass loading was preserved (estimated to contribute with 76% in its original form). Similarly, 24% mass loading of a mixture of Co-oxides (CoO and Co_3_O_4_) were grown on CC substrate, and the HER performance was examined and compared. Figure S11 demonstrated the poor activity of pure CoP/CC and Co_*x*_O_*y*_/CC in comparison with CoP-Co_*x*_O_*y*_/CC, where heterostructure interface is found to be an effective characteristic property in the enhanced HER performance in base.^32,33^

For a better understanding of cathodic hydrogen production kinetics, an essential determination of the reaction mechanism is required by describing the adsorption behavior of intermediate species.^34^ The HER mechanism of the CoP-Co_*x*_O_*y*_/CC (450 °C) electrocatalyst was examined here in 1 M KOH_(aq)_ using the steady-state polarization curves (Tafel) method.^35^ The schematic representation in the Supporting Information (Scheme 1) displays the proposed mechanistic routes at the cathodic catalyst surface of CoP-Co_*x*_O_*y*_/CC under basic electrolyte solutions.

In general, the water electrolysis kinetic pathway varies based on the different reaction conditions. In an alkaline environment, additional energy is required to break the strong covalent H–O–H bond (eq 1); however, an easily reduced coordinate bond in H_3_O^+^ can be achieved simply in acidic medium (eq 2).^15^ The poor reaction kinetics in the base was circumvented here by introducing Co(OH)_2_ to the CoP catalytic system.^36^ While Co(OH)_2_ assists the dissociation of water (eq 3), the nearby CoP electrocatalyst facilitates the adsorption–desorption process of hydrogen intermediates into molecular hydrogen. In our case, Co^2+^ cations are originated from the existing CoO, Co_3_O_4_, and Co_3_(PO_4_)_2_ species in the electrocatalytic system.Volmer reaction in base:
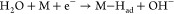
1Volmer reaction in acid:
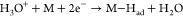
2
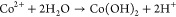
3

Figure S12 displays the calculated Tafel slopes extracted from the corresponding polarization curves for each sample prepared during the phosphatization process at various temperatures. The selected linear curves were fitted by the Tafel equation (eq 4):
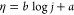
4where η is the overpotential, *j* is the current density, and *b* is the Tafel slope. Tafel slopes of different samples were found to fall within the range of 43.0–59.1 mV dec^–1^ with the smallest measured value for CoP-Co_*x*_O_*y*_/CC (450 °C) in base, proposing Volmer–Heyrovsky HER mechanism at which the desorption step is the rate-determining step as shown in eq 5.Heryrovsky reaction in base:
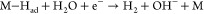
5

Furthermore, the formation of Co(OH)_2_ species during water reduction reaction was confirmed by postelectrocatalytic HR-TEM and XPS analysis. As illustrated in Figure S13, a high population of Co(OH)_2_ on the surface of the electrocatalyst is detected after it has been subjected to the electrolysis process for 10 h in base, which further confirms the proposed mechanism.

One more significant criterion of an excellent electrocatalyst can be expressed in terms of its durability. That was achieved through employing continuous cycling of CoP-Co_*x*_O_*y*_/CC (450 °C) at a scan rate of 50 mV s^–1^ for 100 and 1000 cycles. The HER cathodic current demonstrated in Figure 4d displayed an unchanged LSV curve with minor loss of its catalytic activity. In parallel, long-standing electrochemical stability of as-prepared electrocatalyst was assessed through chronoamperometry (CA) at a constant potential of −72.7 mV vs RHE (Figure S14). The results suggested that CoP-Co_*x*_O_*y*_/CC (450 °C) catalyst can maintain its catalytic activity by retaining 87% of its initial current density for 70 h of continuous operation. Furthermore, the catalytic activity of our catalyst was examined after imperiling to thermal calcination at 300 °C under ambient conditions (Figure 4e). Once more, the catalytically active CoP-Co_*x*_O_*y*_/CC afforded an analogous *i*–*V* curve; consequently, suggesting electrocatalytic efficiency and structural veracity conservation.

We further examined the material preservation through postcatalytic spectroscopic characterizations. Narrow-scan Co 2p and P 2p XPS analysis were used to elucidate the postcatalytic surface and near-surface changes as shown in Figure S13b,c, where no notable shift or disappearance can be discerned in the existing binding energies. The survey spectrum of the postcatalytic specimen shows the presence of Co, P, O, and C, along with K which originated from KOH_(aq)_ electrolyte solution (Figure S13d). Besides, no significant changes in the XRD pattern were detected before and after the electrocatalytic reaction (Figure S15a), indicating the superior stability of CoP-Co_*x*_O_*y*_/CC electrocatalyst. HR-TEM analysis in tandem with electron energy loss spectroscopy (EELS) mapping was also conducted (Figure S15b), further confirming the compositional stability of CoP-Co_*x*_O_*y*_/CC after water electrolysis in base.

It is well-known that electrochemically active sites can play a significant role in HER performances of any electrocatalyst. In order to estimate the active site density of our catalyst and compare it with state-of-the-art Pt/C, underpotential deposition (UPD) technique was applied. Fundamentally, we assume that active sites responsive for H^+^ and Cu^2+^ reduction are the same at an underpotential.^16^ As a result, the exchange of copper charges during oxidative stripping attained in UPD can be used for active site approximation. Figure 4f displays the current–voltage (CV) scan of CoP-Co_*x*_O_*y*_/CC in different solutions. The black CV curve was measured in 0.5 M H_2_SO_4(aq)_ solution and considered as a baseline at which no oxidation or reduction peaks were observed. However, a single reversible oxidation–reduction peak was detected when the scan was taken in a solution of 0.5 M H_2_SO_4(aq)_ and 20 mM CuSO_4(aq)_ (red curve), which is due to an overlap of copper under- and overpotential deposition (OPD). By adding NaCl_(aq)_ to the previously mentioned solution, UPD and OPD regions, along with their stripping, were clearly shown as displayed in the blue scan. CV scans were also observed for 2 mg cm^–2^ Pt–C/CC electrocatalyst (Figure S16). Stripping of deposited UPD Cu was carried out at different overpotential ranging from 0.05 to −0.12 V vs Ag/AgCl (see Figure S17a,b for details), and the required charges to strip the deposited UPD Cu within the same overpotential range were plotted for both CoP-Co_*x*_O_*y*_/CC and Pt–C/CC catalysts (Figure 4g). Evaluating the charge quantity at the plateau enables us to estimate the density of the active site of our catalyst as follows (eqs 6 and 7):
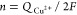
6
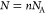
7where *n*, *Q*_Cu_^2+^, *F*, *N*, and *N*_A_ are the number of moles, the charge required to strip the UPD Cu, the Faraday constant (96 485.3329 C mol^–1^), number of active sites, and Avogadro’s number (6.022 × 10^23^ mol^–1^), respectively. For a 1 cm^2^ electrode surface area, active site densities were estimated for both CoP-Co_*x*_O_*y*_/CC and Pt–C/CC as 12.7 × 10^17^ and 32.4 × 10^17^ sites cm^–2^, respectively. Furthermore, UPD Cu elemental distribution on CoP-Co_*x*_O_*y*_/CC and Pt–C/CC was tested using scanning electron microscopy (STEM) equipped with EELS (see Figures 4h and S18 for details). For each catalyst, it was observed that Cu is homogeneously distributed all over the selected range of vision. Additionally, the electrochemical active surface area of CoP electrode prepared at different temperatures was estimated by measuring the capacitances at the interfacial area between the electrode surface and electrolyte solution. Cyclic voltammograms in the region between −0.9 and −0.7 V vs RHE of CoP_*x*_ /CC phosphatized at various temperatures were collected (see Figure S19 for details). The current density differences (Δ*J* = *J*_a_ – *J*_c_) were taken at −0.9 V vs RHE and plotted with respect to the corresponding scan rate. Double-layer capacitance (*C*_dl_) was then calculated from the linear plots (Figure S20). On comparing of all the CoP_*x*_ electrocatalysts prepared at various temperatures, the CV curves show that CoP-Co_*x*_O_*y*_/CC formed at 450 °C can provide a higher anodic and cathodic current densities; consequently, the highest surface area and roughness which is associated with the excellent HER efficiency.

Afterward, the intrinsic catalytic activity was investigated through TOF quantification at an overpotential of 100 mV vs RHE. Significantly, the as-prepared CoP-Co_*x*_O_*y*_/CC HER electrocatalyst unveiled a high TOF of about 0.6 H_2_ s^–1^, comparable to that of state-of-the-art Pt–C/CC (∼1 H_2_ s^–1^) (Figure S21). These outstanding features may play an essential role in accelerating charge transfer throughout the electrocatalytic reaction, which could be another reason for the excellent HER activity compared with other CoP-based electrocatalysts.

## Conclusions

In summary, this study reports a facile method to synthesize highly durable CoP-Co_*x*_O_*y*_/CC heterostructured composites for driving the HER in base. The method involves the deposition of Co-oxy species on CC supporting substrate followed by vapor phase phosphatization carried out in a modified CVD system by using elemental red phosphorus precursors. The effect of the phosphatization temperatures on the formation of different phases of cobalt phosphide was investigated in detail. Structural and chemical composition analyses after phosphatization reveal the formation of a CoP-Co_*x*_O_*y*_ heterostructured composite, orthorhombic CoP, and monoclinic CoP_2_ at different temperatures. Electrochemical tests alongside detailed studies of the electrocatalytic activities of materials with different surface compositions indicate that the CoP-Co_*x*_O_*y*_/CC electrocatalyst obtained after phosphatization to 450 °C is the most active HER electrocatalyst in the whole series. It can deliver a catalytic current density of 10 mA cm^–2^ at one of the lowest overpotentials of −43 mV ever reported in alkaline water. Moreover, its initial current density is retained for 1000 cycles, with only a minor loss in its catalytic activity alongside with a high TOF of 0.6 H_2_ s^–1^ at an overpotential of 100 mV vs RHE. DFT calculations provide a systematic understanding of the experimental findings. The proposed low-cost and highly durable electrocatalyst shows promise for larger-scale hydrogen production through water electrolysis.

## Experimental Section

### Materials

All chemicals including cobalt(II) nitrate hexahydrate (Co(NO_3_)_2_.6H_2_O, ≥96%), potassium hydroxide (KOH_(aq)_, ≥85%), copper(II) sulfate pentahydrate (CuSO_4_.5H_2_O, ≥98%), sodium chloride (NaCl, ≥99%), platinum (Pt, nominally 20% on carbon black), red phosphorus (P, ≥99.99%), and ethanol (≥85%) were purchased from Sigma-Aldrich and used without further purification. Water used was purified through a Millipore ultrapure water system (18.2 MΩ.cm at 25 °C).

### Characterizations

The morphology and EELS of the catalysts was studied by field-emission scanning electron microscopy (FESEM, FEI Quanta 600). XRD (Bruker D8 Discover diffractometer, using Cu Kα radiation, λ = 1.54 Å) was used at 2θ range of 20–80° to investigate the phase composition. Temperature-dependent XRD (Bruker D8 Advance nonambient temperature) was employed for phase identification at various temperatures (650–850 °C) in a vacuum with a heating rate of 15 °C min^–1^ and delay time of 3 min prior measurement for each temperature step. Thermogravimetric (TG) analysis was carried out using a simultaneous thermal analyzer (STA, STA 449 F1 Netzsch) in the N_2(g)_ atmosphere with a heating rate of 15 °C min^–1^. The lattice structure was studied by transmission electron microscopy (FEI Titan ST, operated at 300 kV), while electronic structure and surface/near-surface composition is studied by XPS using a Kratos Axis Ultra DLD spectrometer equipped with a monochromatic Al Kα X-ray source (*hν* = 1486.6 eV) operating at 150 W, a multichannel plate, and a delay line detector at about 1.0 x10^–9^ Torr background pressure. Measurements were carried out at a 0° takeoff angle (angle between the sample surface normal and the electron optical axis of the spectrometer). All spectra were recorded using an aperture slot of 300 μm × 700 μm. The survey and high-resolution spectra were collected at fixed analyzer pass energies of 160 and 20 eV, respectively. Samples were mounted in a floating mode in order to avoid differential charging. Charge neutralization was carried out for all samples. Binding energies were referenced to the C 1s peak (set at 284.4 eV) of the sp^2^-hybridized (C=C) carbon from the carbon cloth substrate. The data were analyzed with the commercially available CasaXPS software. The individual peaks were fitted by a Gaussian (70%)–Lorentzian (30%) (GL30) function after a linear or Shirley-type background subtraction.

### Electrochemical Measurements

Electrochemical measurements were carried out on a Metrohm PGSTAT 302N Autolab Potentiostat at room temperature. The hydrogen evolution reaction performance of all catalysts was assessed by measuring polarization curves with linear sweep voltammetry (LSV) at a scan rate of 0.1 mV/s. In 1 M KOH_(aq)_ (pH 13.95) solution, the Nernst equation becomes *E*(RHE) = *E*(Ag/AgCl) + 1.04005 V. In a hydrogen-saturated electrolyte, a separate RHE calibration was carried out with a 1.041244 V offset, which perfectly coincides with 1.04005 in the equation. A graphite rod was used as a counter electrode, while Ag/AgCl (in 3 M KCl_(aq)_ solution) electrode was used as a reference electrode.

### Cathode Preparation

CC conductive material with a dimension of 1.5 × 1 cm^2^ was used for the electrochemical deposition process. Prior to its use, CC was washed with ethanol and deionized water. Next, a geometrical area of 1 cm^2^ was immersed into 0.1 M Co(NO_3_)_2(aq)_ solution. Pt foil was utilized as a counter electrode in the electrochemical cell, while a Ag/AgCl (in 3 M KCl_(aq)_ solution) electrode was used as a reference electrode. The electrodeposition process was conducted at constant current (−10 mA cm^–2^) for 40 min and left overnight under air exposure. Later, the electrochemically deposited electrode was fed into a close-ended tube secured inside a tube furnace along with red phosphorus precursor for phosphatization process. The tube furnace was pumped initially and purged with Ar (60 sccm) and H_2_ (20 sccm) for 30 min (∼10 Torrs) to exclude oxygen and humidity from the system. The phosphatization reaction was carried out under vacuum for 30 min interval of time and with a heating rate of 15 °C min^–1^ at several temperatures ranging between 450 to 850 °C. The as-prepared catalyst was then rinsed with 0.5 M H_2_SO_4(aq)_ solution and Milli-Q water for impurities removal. The areal density of the as-prepared material was determined, after drying in a vacuum oven, to be ∼10 mg cm^–2^ by a high-accuracy weighing balance (see Table S4 for details).

### Calculations of Normalized Current Densities

The current densities normalized by electrochemical active surface area (ECSA) was calculated from the corresponding current using the following equation:
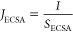
8where *J*_ECSA_ is the current density normalized by ECSA, *I* is the current (mA), and *S*_ECSA_ = *C*_dl_/*C*_s_ (where *C*_dl_ represents the double layer capacitance and *C*_s_ = 0.040 mF cm^–2^).^37^

### Active Site Density

Copper UPD experiments were carried out in 0.5 M H_2_SO_4(aq)_, 20 mM CuSO_4(aq)_, and 60 mM NaCl_(aq)_ initiated with electrochemical cleaning at 0.5 V vs Ag/AgCl for 100 s. Within the same solution, copper deposition was accomplished at different underpotentials (0.05 to −0.12 V vs Ag/AgCl) for 120 s followed by linear sweep voltammetry (LSV) with a scan speed of 0.002 V s^–1^.

### Calculation of Turnover Frequency (TOF)

The HER TOF per site for our catalyst was calculated at η = 100 mV by applying the following formula:
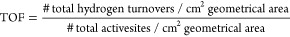
9where the numerator was calculated as follows:
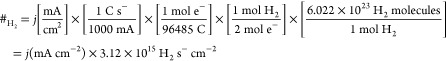
And the denominator of eq 9 was obtained from the previously estimated active site density.

### Reference Electrode Calibration

Prior to the measurements, the 1 M KOH_(aq)_ electrolyte solution was purged with hydrogen gas for 30 min. Pt wires and Ag/AgCl (in 3 M KCl_(aq)_ solution) were used as a counter, working, and reference electrodes, respectively. Current–voltage (CV) curves were achieved at a scan rate of 5 mV s^–1^. The thermodynamic potential of the hydrogen electrode reactions was then taken at the zero current crossings the average of the two potentials (Figure S1). The result shows that *E*(Ag/AgCl) is lower than *E*(RHE) by 1.041244 V.

## Supporting Information Available

Details of materials, characterizations studies, electrode preparations, and computational studies, additional characterizations include XRD, TGA, STEM, EDX, SEM, XPS, and CV polarization curves and schematics of mechanism for HER performance, and results of DFT studies for HER (PDF)

## References

[ref1] Chu S.; Majumdar A. Opportunities and Challenges for a Sustainable Energy Future. Nat. 2012, 488 (7411), 294–303. 10.1038/nature11475. 22895334

[ref2] Liu Y.; Li X.; Zhang Q. H.; Li W. D.; Xie Y.; Liu H. Y.; Shang L.; Liu Z. Y.; Chen Z. M.; Gu L.; Tang Z. Y.; Zhang T. R.; Lu S. Y. A General Route to Prepare Low-Ruthenium-Content Bimetallic Electrocatalysts for pH-Universal Hydrogen Evolution Reaction by Using Carbon Quantum Dots. Angew. Chem., Int. Ed. 2020, 59 (4), 1718–1726. 10.1002/anie.201913910. 31799763

[ref3] Hu C. L.; Zhang L.; Gong J. L. Recent Progress Made in the Mechanism Comprehension and Design of Electrocatalysts for Alkaline Water Splitting. Energy Environ. Sci. 2019, 12 (9), 2620–2645. 10.1039/C9EE01202H.

[ref4] Mahmood N.; Yao Y. D.; Zhang J. W.; Pan L.; Zhang X. W.; Zou J. J. Electrocatalysts for Hydrogen Evolution in Alkaline Electrolytes: Mechanisms, Challenges, and Prospective Solutions. Adv. Sci. 2018, 5 (2), 1700464–1700487. 10.1002/advs.201700464. PMC5827647 29610722

[ref5] Men Y.; Li P.; Zhou J.; Chen S.; Luo W. Trends in Alkaline Hydrogen Evolution Activity on Cobalt Phosphide Electrocatalysts Doped with Transition Metals. Phys. Sci. 2020, 1, 100136–100151. 10.1016/j.xcrp.2020.100136.

[ref6] Zhu J.; Hu L. S.; Zhao P. X.; Lee L. Y. S.; Wong K. Y. Recent Advances in Electrocatalytic Hydrogen Evolution Using Nanoparticles. Chem. Rev. 2020, 120 (2), 851–918. 10.1021/acs.chemrev.9b00248. 31657904

[ref7] Zhang J. Q.; Zhao Y. F.; Guo X.; Chen C.; Dong C. L.; Liu R. S.; Han C. P.; Li Y. D.; Gogotsi Y.; Wang G. X. Single Platinum Atoms Immobilized on an MXene as an Efficient Catalyst for the Hydrogen Evolution Reaction. Nat. Catal 2018, 1 (12), 985–992. 10.1038/s41929-018-0195-1.

[ref8] Wang P. T.; Jiang K. Z.; Wang G. M.; Yao J. L.; Huang X. Q. Phase and Interface Engineering of Platinum-Nickel Nanowires for Efficient Electrochemical Hydrogen Evolution. Angew. Chem., Int. Ed. 2016, 55 (41), 12859–12863. 10.1002/anie.201606290. 27629828

[ref9] Bao M. J.; Amiinu I. S.; Peng T.; Li W. Q.; Liu S. J.; Wang Z.; Pu Z. H.; He D. P.; Xiong Y. L.; Mu S. C. Surface Evolution of PtCu Alloy Shell over Pd Nanocrystals Leads to Superior Hydrogen Evolution and Oxygen Reduction Reactions. ACS Energy Lett. 2018, 3 (4), 940–945. 10.1021/acsenergylett.8b00330.

[ref10] Zheng Y.; Jiao Y.; Zhu Y. H.; Li L. H.; Han Y.; Chen Y.; Du A. J.; Jaroniec M.; Qiao S. Z. Hydrogen Evolution by a Metal-Free Electrocatalyst. Nat. Commun. 2014, 5, 4783–4791. 10.1038/ncomms4783. 24769657

[ref11] Pan Y.; Lin Y.; Chen Y. J.; Liu Y. Q.; Liu C. G. Cobalt Phosphide-Based Electrocatalysts: Synthesis and Phase Catalytic Activity Comparison for Hydrogen Evolution. J. Mater. Chem. A 2016, 4 (13), 4745–4754. 10.1039/C6TA00575F.

[ref12] Alsabban M. M.; Yang X. L.; Wahyudi W.; Fu J. H.; Hedhili M. N.; Ming J.; Yang C. W.; Nadeem M. A.; Idriss H.; Lai Z. P.; Li L. J.; Tung V.; Huang K. W. Design and Mechanistic Study of Highly Durable Carbon-Coated Cobalt Diphosphide Core-Shell Nanostructure Electrocatalysts for the Efficient and Stable Oxygen Evolution Reaction. ACS Appl. Mater. Interfaces 2019, 11 (23), 20752–20761. 10.1021/acsami.9b01847. 31091878

[ref13] Zhang Y.; Gao L.; Hensen E. J. M.; Hofmann J. P. Evaluating the Stability of Co_2_P Electrocatalysts in the Hydrogen Evolution Reaction for Both Acidic and Alkaline Electrolytes. ACS Energy Lett. 2018, 3 (6), 1360–1365. 10.1021/acsenergylett.8b00514. 29911183 PMC5996345

[ref14] Mishra I. K.; Zhou H. Q.; Sun J. Y.; Qin F.; Dahal K.; Bao J. M.; Chen S.; Ren Z. F. Hierarchical CoP/Ni_5_P_4_/CoP Microsheet Arrays as a Robust pH-Universal Electrocatalyst for Efficient Hydrogen Generation. Energy Environ. Sci. 2018, 11 (8), 2246–2252. 10.1039/C8EE01270A.

[ref15] Li X.; Liu P. F.; Zhang L.; Zu M. Y.; Yang Y. X.; Yang H. G. Enhancing Alkaline Hydrogen Evolution Reaction Activity through Ni-Mn_3_O_4_ Nanocomposites. Chem. Commun. 2016, 52 (69), 10566–10569. 10.1039/C6CC04141H. 27500290

[ref16] Yang X. L.; Lu A. Y.; Zhu Y.; Min S. X.; Hedhili M. N.; Han Y.; Huang K. W.; Li L. J. Rugae-like FeP Nanocrystal Assembly on a Carbon Cloth: an Exceptionally Efficient and Stable Cathode for Hydrogen Evolution. Nanoscale 2015, 7 (25), 10974–10981. 10.1039/C5NR02375K. 26058361

[ref17] Yang X. L.; Lu A. Y.; Zhu Y. H.; Hedhili M. N.; Min S. X.; Huang K. W.; Han Y.; Li L. J. CoP Nanosheet Assembly Grown on Carbon Cloth: A Highly Efficient Electrocatalyst for Hydrogen Generation. Nano Energy 2015, 15, 634–641. 10.1016/j.nanoen.2015.05.026.

[ref18] Feng L. G.; Vrubel H.; Bensimon M.; Hu X. L. Easily-Prepared Dinickel Phosphide (Ni_2_P) Nanoparticles as an Efficient and Robust Electrocatalyst for Hydrogen Evolution. Phys. Chem. Chem. Phys. 2014, 16 (13), 5917–5921. 10.1039/c4cp00482e. 24554088

[ref19] Chen Y. C.; Lu A. Y.; Lu P.; Yang X. L.; Jiang C. M.; Mariano M.; Kaehr B.; Lin O.; Taylor A.; Sharp I. D.; Li L. J.; Chou S. S.; Tung V. Structurally Deformed MoS_2_ for Electrochemically Stable, Thermally Resistant, and Highly Efficient Hydrogen Evolution Reaction. Adv. Mater. 2017, 29 (44), 1703863–1703874. 10.1002/adma.201703863. 29024072

[ref20] Zhang R.; Wang X. X.; Yu S. J.; Wen T.; Zhu X. W.; Yang F. X.; Sun X. N.; Wang X. K.; Hu W. P. Ternary NiCo_2_P_x_ Nanowires as pH-Universal Electrocatalysts for Highly Efficient Hydrogen Evolution Reaction. Adv. Mater. 2017, 29 (9), 1605502–1605508. 10.1002/adma.201605502. 28028846

[ref21] Su L.; Cui X. Z.; He T.; Zeng L. M.; Tian H.; Song Y. L.; Qi K.; Xia B. Y. Surface Reconstruction of Cobalt Phosphide Nanosheets by Electrochemical Activation for Enhanced Hydrogen Evolution in Alkaline Solution. Chem. Sci. 2019, 10 (7), 2019–2024. 10.1039/C8SC04589E. 30842859 PMC6375356

[ref22] Zhao X. X.; Fan Y. P.; Wang H. Y.; Gao C. Y.; Liu Z. Y.; Li B. J.; Peng Z. K.; Yang J. H.; Liu B. Z. Cobalt Phosphide-Embedded Reduced Graphene Oxide as a Bifunctional Catalyst for Overall Water Splitting. Acs Omega 2020, 5 (12), 6516–6522. 10.1021/acsomega.9b04143. 32258887 PMC7114733

[ref23] Zhu Y. P.; Liu Y. P.; Ren T. Z.; Yuan Z. Y. Self-Supported Cobalt Phosphide Mesoporous Nanorod Arrays: A Flexible and Bifunctional Electrode for Highly Active Electrocatalytic Water Reduction and Oxidation. Adv. Funct. Mater. 2015, 25 (47), 7337–7347. 10.1002/adfm.201503666.

[ref24] Ishida K.; Nishizawa T. The Co-P (Cobalt-Phosphorus) System. Bull. Alloy Phase Diagrams. 1990, 11 (6), 555–560. 10.1007/BF02841716.

[ref25] Alsabban M. M.; Min S. X.; Hedhili M. N.; Ming J.; Li L. J.; Huang K. W. Growth of Layered WS_2_ Electrocatalysts for Highly Efficient Hydrogen Production Reaction. ECS J. Solid State Sci. Technol. 2016, 5 (11), Q3067–Q3071. 10.1149/2.0141611jss.

[ref26] Biesinger M. C.; Payne B. P.; Grosvenor A. P.; Lau L. W. M.; Gerson A. R.; Smart R. S. Resolving Surface Chemical States in XPS Analysis of First Row Transition Metals, Oxides and Hydroxides: Cr, Mn, Fe, Co and Ni. Appl. Surf. Sci. 2011, 257 (7), 2717–2730. 10.1016/j.apsusc.2010.10.051.

[ref27] Callejas J. F.; Read C. G.; Popczun E. J.; McEnaney J. M.; Schaak R. E. Nanostructured Co_2_P Electrocatalyst for the Hydrogen Evolution Reaction and Direct Comparison with Morphologically Equivalent CoP. Chem. Mater. 2015, 27 (10), 3769–3774. 10.1021/acs.chemmater.5b01284.

[ref28] Madhu R.; Veeramani V.; Chen S. M.; Manikandan A.; Lo A. Y.; Chueh Y. L. Honeycomb-like Porous Carbon-Cobalt Oxide Nanocomposite for High-Performance Enzyme less Glucose Sensor and Supercapacitor Applications. ACS Appl. Mater. Interfaces 2015, 7 (29), 15812–15820. 10.1021/acsami.5b04132. 26125456

[ref29] Arunachalam P.; Shaddad M. N.; Alamoudi A. S.; Ghanem M. A.; Al-Mayouf A. M. Microwave-Assisted Synthesis of Co_3_(PO_4_)_2_ Nanospheres for Electrocatalytic Oxidation of Methanol in Alkaline Media. Catalysts. 2017, 7 (4), 119–130. 10.3390/catal7040119.

[ref30] Cobo S.; Heidkamp J.; Jacques P. A.; Fize J.; Fourmond V.; Guetaz L.; Jousselme B.; Ivanova V.; Dau H.; Palacin S.; Fontecave M.; Artero V. A Janus Cobalt-Based Catalytic Material for Electro-Splitting of Water. Nat. Mater. 2012, 11 (9), 802–807. 10.1038/nmat3385. 22863815

[ref31] Chang J. F.; Xiao Y.; Xiao M. L.; Ge J. J.; Liu C. P.; Xing W. Surface Oxidized Cobalt-Phosphide Nanorods As an Advanced Oxygen Evolution Catalyst in Alkaline Solution. ACS Catal. 2015, 5 (11), 6874–6878. 10.1021/acscatal.5b02076.

[ref32] Jiao M.; Chen Z.; Zhang X.; Mou K.; Liu L. Multicomponent N doped graphene coating Co@Zn heterostructures electrocatalysts as high efficiency HER electrocatalyst in alkaline electrolyte. Int. J. Hydrogen Energy 2020, 45 (33), 16326–16336. 10.1016/j.ijhydene.2020.04.121.

[ref33] Hu X.; Zhang S.; Sun J.; Yu L.; Qian X.; Hu R.; Wang Y.; Zhao H.; Zhu J. 2D Fe-containing cobalt phosphide/cobalt oxide lateral heterostructure with enhanced activity for oxygen evolution reaction. Nano Energy 2019, 56, 109–117. 10.1016/j.nanoen.2018.11.047.

[ref34] Conway B. E.; Bai L. Determination of the Adsorption Behavior of Overpotential-Deposited Hydrogen-Atom Species in the Cathodic Hydrogen-Evolution Reaction by Analysis of Potential-Relaxation Transients. J. Chem. Soc. Faraday Trans. I 1985, 81, 1841–1862. 10.1039/f19858101841.

[ref35] Azizi O.; Jafarian M.; Gobal F.; Heli H.; Mahjani M. G. The Investigation of the Kinetics and Mechanism of Hydrogen Evolution Reaction on Tin. Int. J. Hydrogen Energy 2007, 32 (12), 1755–1761. 10.1016/j.ijhydene.2006.08.043.

[ref36] Sayeed M. A.; Herd T.; O'Mullane A. P. Direct electrochemical formation of nanostructured amorphous Co(OH)_2_ on gold electrodes with enhanced activity for the oxygen evolution reaction. J. Mater. Chem. A 2016, 4, 991–999. 10.1039/C5TA09125J.

[ref37] Luan C.; Liu G.; Liu Y.; Yu L.; Wang Y.; Xiao Y.; Qiao H.; Dai X.; Zhang X. Structure Effects of 2D Materials on α-Nickel Hydroxide for Oxygen Evolution Reaction. ACS Nano 2018, 12 (4), 3875–3885. 10.1021/acsnano.8b01296. 29630354

