# Supplementary material for: Unusual Activity of Rationally Designed Cobalt Phosphide/Oxide Heterostructure Composite for Hydrogen Production in Alkaline Medium
Source: ACS Nano. 2022 Mar 7;16(3):3906–16. doi: 10.1021/acsnano.1c09254 (PMC8945697; doi:10.1021/acsnano.1c09254)
Supplement: Supplementary file 1 — nn1c09254_si_001.pdf [file nn1c09254_si_001.pdf]

Unusual Activity of Rationally Designed Cobalt Phosphide/Oxide Heterostructure Composite for Hydrogen Production in Alkaline Medium

Merfat M. Alsabban[1,2,3], Mathan Kumar Eswaran[1], Karthik Peramaiah[1,2], Wandi Wahyudi[1], Xiulin Yang[1,2], Vinoth Ramalingam[1,2], Mohamed. N. Hedhili[4], Xiaohe Miao[4], Udo Schwingenschlögl[1], Lain-Jong Li[1,5,]* Vincent Tung[1]* and Kuo-Wei Huang[1,2]*

[1]Division of Physical Sciences and Engineering, King Abdullah University of Science and Technology, Thuwal, 23955-6900, Kingdom of Saudi Arabia.

[2]KAUST Catalysis Center, King Abdullah University of Science and Technology, Thuwal, 23955-6900, Kingdom of Saudi Arabia.

[3]Department of Chemistry, University of Jeddah, Jeddah, 21959, Kingdom of Saudi Arabia.

[4]Core Labs, King Abdullah University of Science and Technology, Thuwal, 23955-6900, Kingdom of Saudi Arabia.

[5]Department of Mechanical Engineering, The University of Hong Kong, Pokfulam Road, Hong Kong.

E-mail: lanceli1@hku.hk (L.-J. L.); vincent.tung@kaust.edu.sa (V.T.); hkw@kaust.edu.sa (K.-W.H.)

Computational Details

Spin-polarized density functional theory is used as implemented in the Vienna ab-initio simulation package (projector augmented wave method) with a plane wave energy cutoff of 550 eV and a 5× 5×1 Monkhorst-Pack k-grid.[1, 2] The exchange-correlation potential is treated in the Perdew-Burke-Ernzerhof generalized gradient approximation with van der Waals corrections.[3] The total energy changes and maximum atomic force are converged to 10^{-5} eV and 0.001 eV $Å^{-1}$, respectively. Following the experimental setup, we model the (110) direction of CoP, CoO, and Co_3O_4. The relaxed lattice parameters of the unit cell of CoP turn out to be a = 3.226 Å, b = 5.015 Å, c = 5.505 Å, in agreement with previous experimental and theoretical studies.[4] We extract a monolayer of CoP (110) and obtain relaxed lattice parameters of a = 5.501 Å and b = 5.879 Å. The relaxed lattice parameters of the primitive cells of CoO and Co_3O_4 turn out to be a = b = c = 2.986 and 5.713 Å, respectively, in agreement with previous experimental and theoretical studies.[5] We extract a monolayer of CoO (110) in a 2 × 2 × 1 supercell and obtain relaxed lattice parameters of a = b = 5.478 Å. We extract a monolayer of Co_3O_4 (100) and obtain relaxed lattice parameters of a = 5.629 Å and b = 5.617 Å.

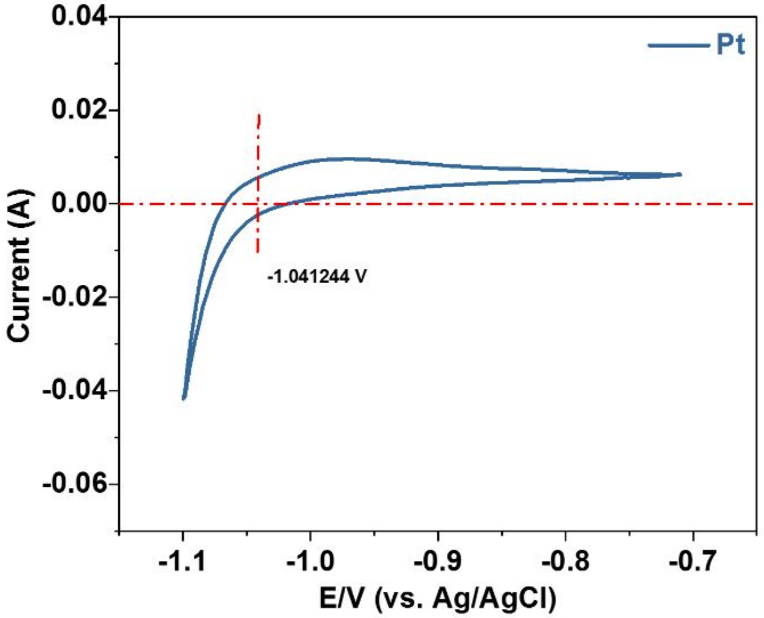

Figure S1. RHE voltage calibration. The calibration was performed in the high purity hydrogen saturated electrolyte with a Pt wire as a working electrode. The current-voltage scans were run at a scan rate of 10 mV s^{-1}. The average of the two potentials at which the current crossed zero was taken to be the thermodynamic potential for the oxygen electrode reactions. In 1 M $KOH_{(aq)}$ (pH = 13.95) solution, Nernst equation becomes E(RHE) = E(Ag/AgCl) + 1.04005. A separate RHE calibration in a hydrogen saturated electrolyte has been accomplished with 1.041244 V offset, which perfectly coincides with 1.04005 in the equation.

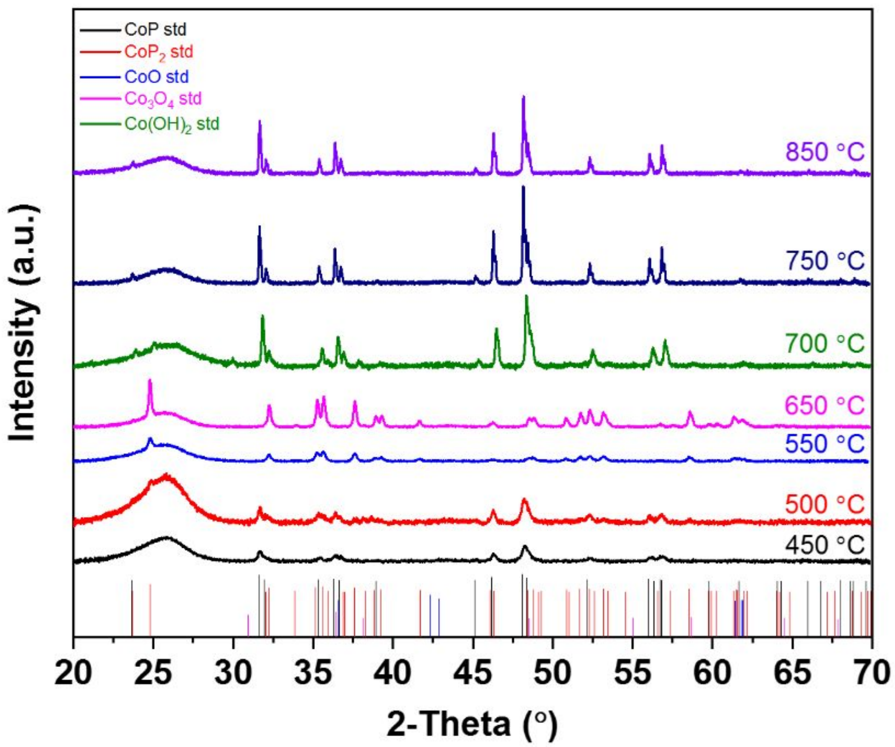

Figure S2. XRD patterns of different phases formed at different phosphatization temperatures. CoP-Co_xO_y/CC formed at 450 °C, CoP_2/CC formed at 550 °C and 650 °C, CoP/CC formed at 750 °C and 850 °C, and CoP-CoP_2/CC multiphase formed at 500 °C and 700 °C.

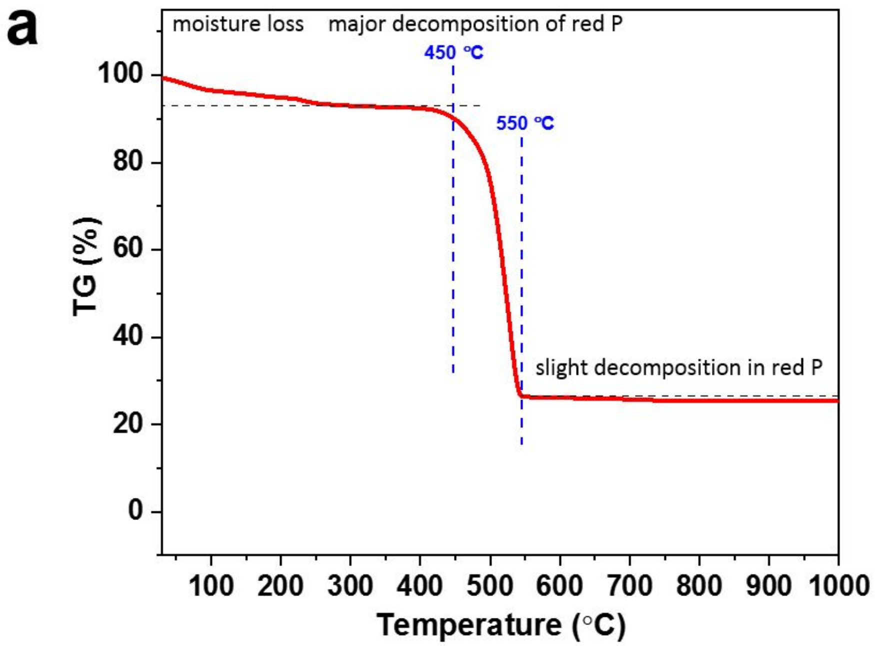

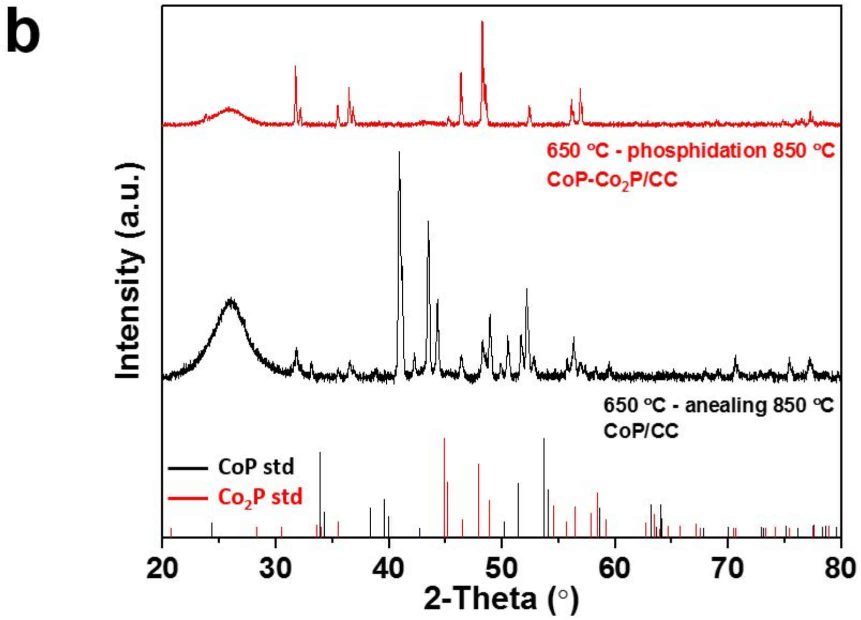

Figure S3. (a) TGA analysis for elemental red phosphorous. (b) XRD spectra of annealed and phosphatized CoP_2/CC to 850 °C.

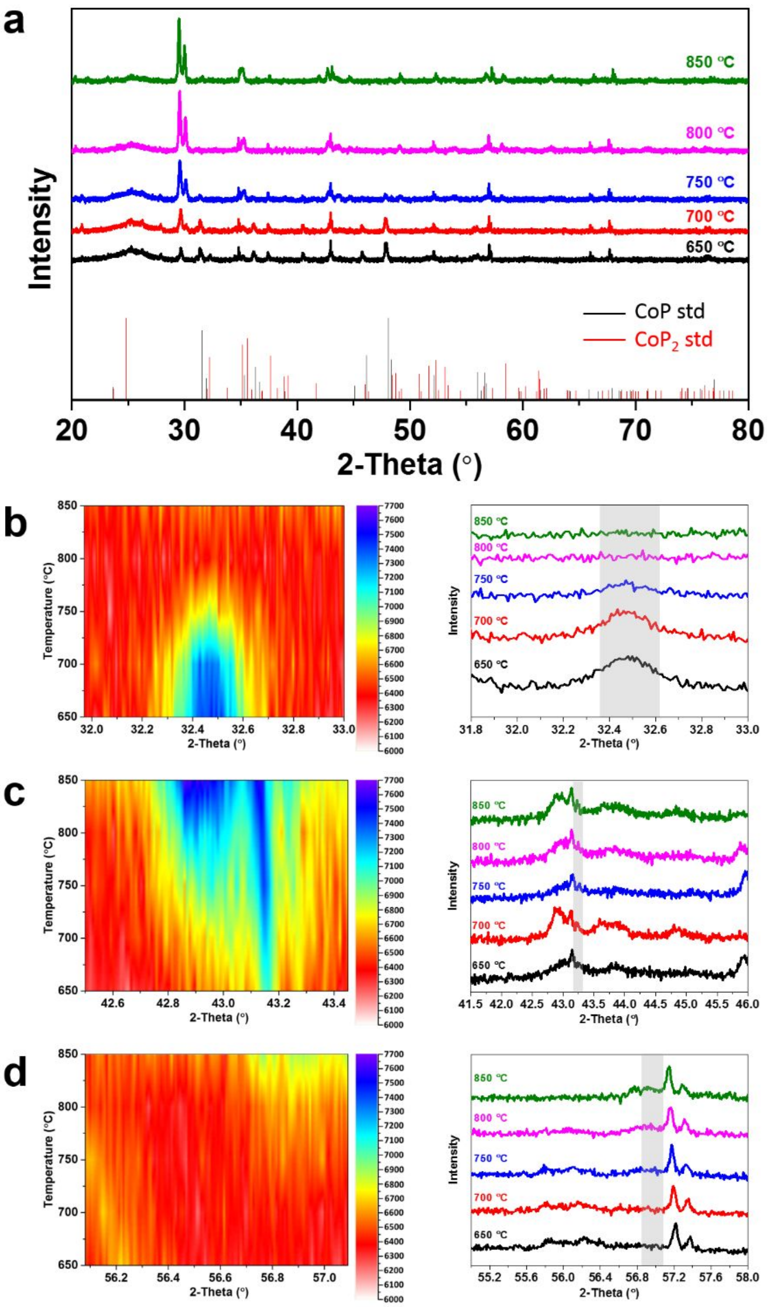

Figure S4. (a) 1D temperature dependent XRD scans of CoP_2/CC (650 °C) upon heating from 650 °C to 850 °C. (b) Enlarged (020) peak (2θ = 31.8° – 33.0°) and its corresponding 2D XRD pattern revealing CoP_2 decomposition at high temperatures. (c) Enlarged (211) peak (2θ = 41.5° – 46.0°) and its corresponding 2D XRD pattern revealing Co_2P formation as temperature elevating. (d) Enlarged (301) peak (2θ = 55.0° – 58.0°) and its corresponding 2D XRD pattern revealing CoP formation as temperature elevating.

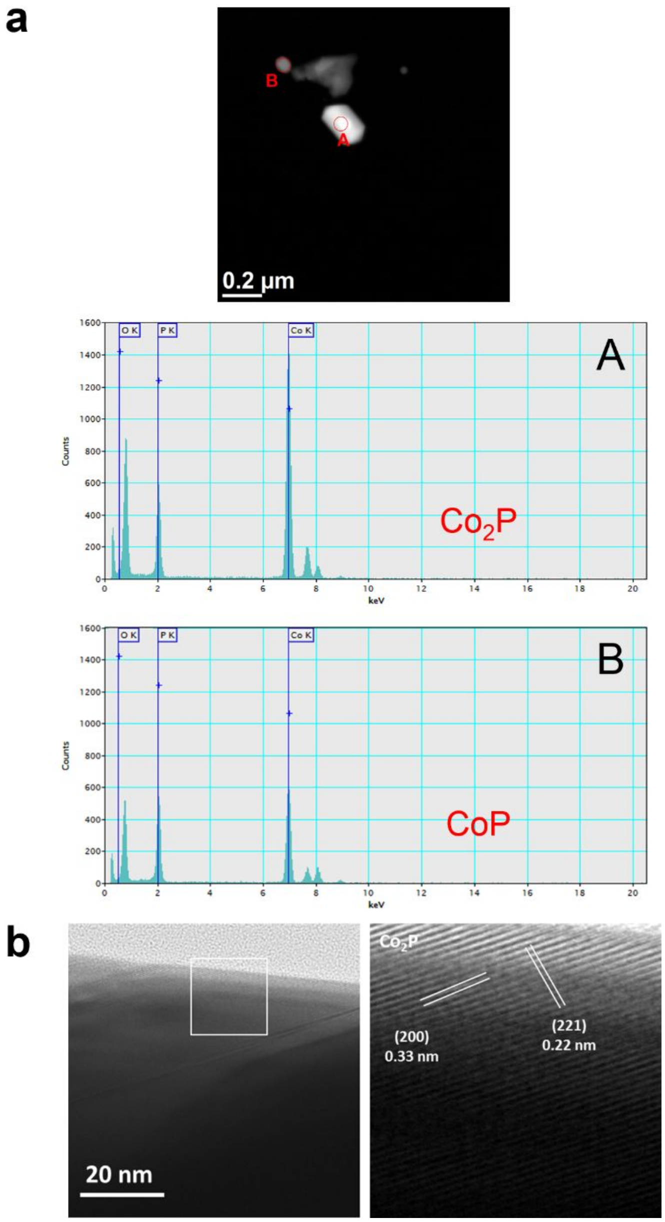

Figure S5. (a) STEM image and the corresponding EDX elemental composition of Co_2P/CC and CoP/CC formed through annealing of CoP_2/CC (650 °C) to 850 °C. (b) HRTEM imaging of Co_2P/CC formed through annealing of CoP_2/CC to 850 °C.

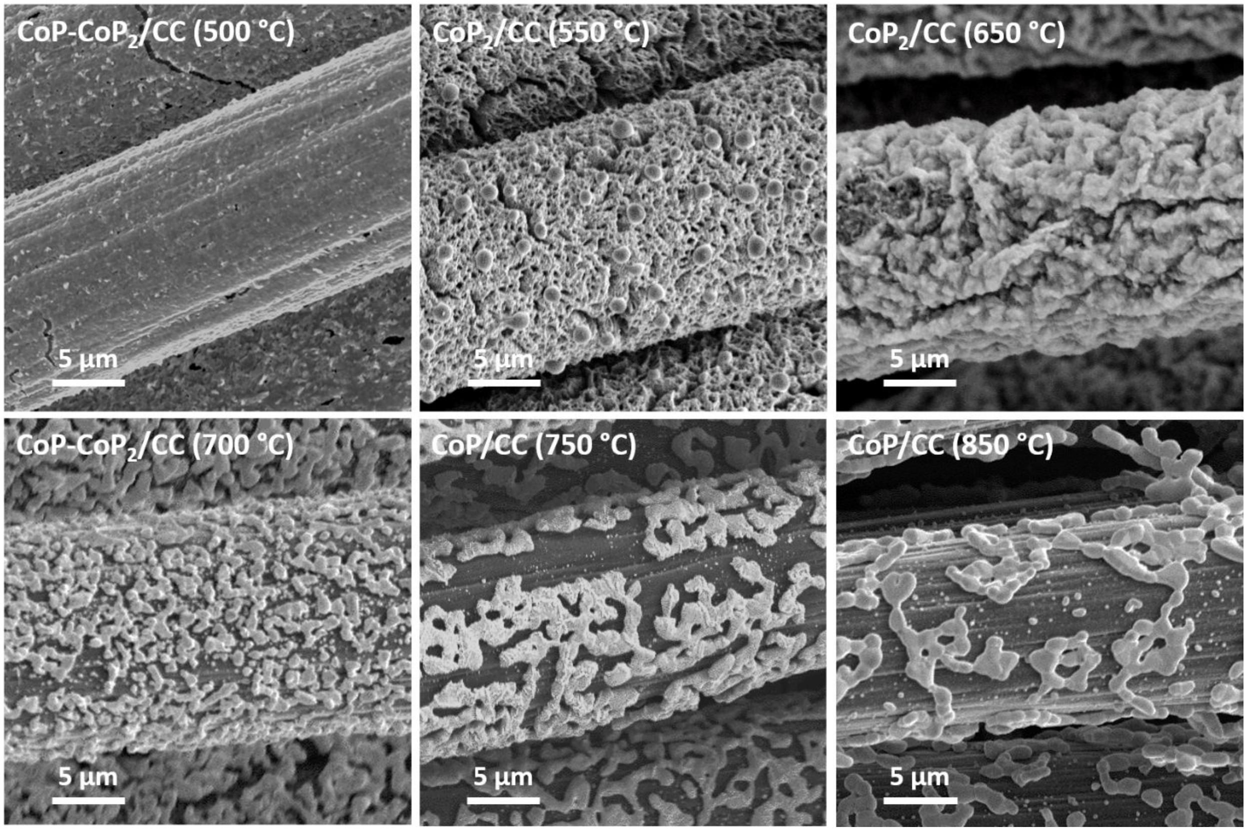

Figure S6. SEM images of CoP_x/CC (5 µm diameter) formed at different temperatures during phosphatization process.

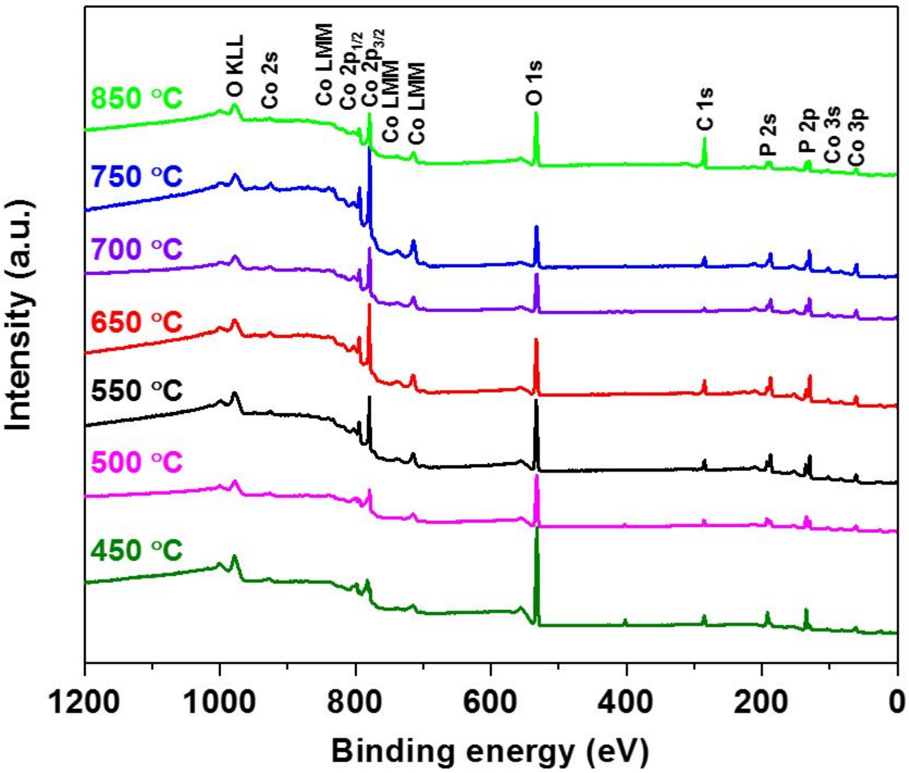

Intensity (a.u.)
Binding energy (eV)
850 °C
750 °C
700 °C
650 °C
550 °C
500 °C
450 °C
O KLL
Co 2s
Co LMM
Co 2p1/2
Co 2p3/2
Co LMM
Co LMM
O 1s
C 1s
P 2s
P 2p
Co 3s
Co 3p
1200
1000
800
600
400
200
0

Figure S7. Survey spectrum of CoP-Co_xO_y/CC (450 °C) and CoP_x/CC formed at different phosphatization temperatures

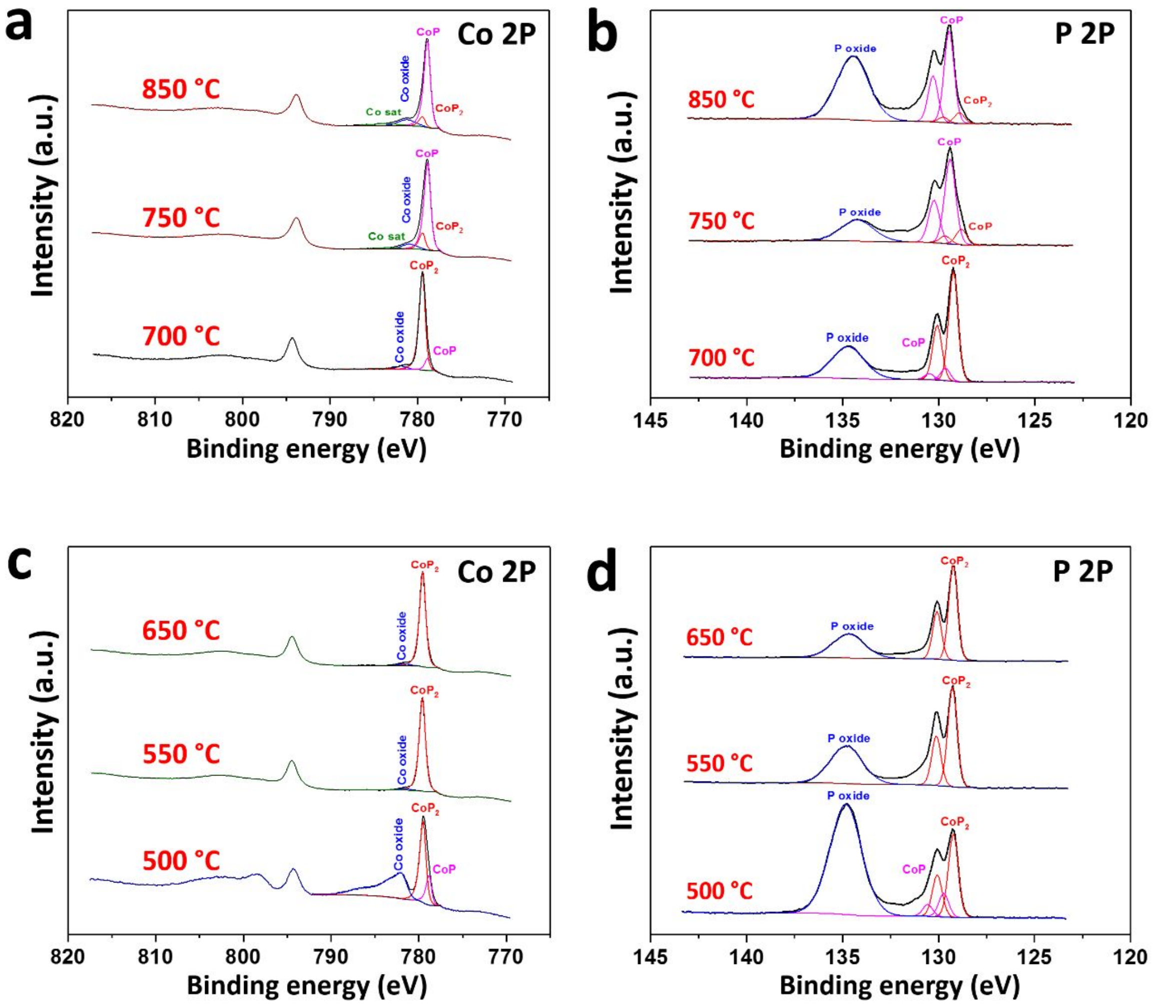

Figure S8. XPS spectra for: (a,c) Co 2p of CoP_x /CC formed at different phosphatization temperatures. (b,d) P 2p of CoP_x /CC formed at different phosphatization temperatures.

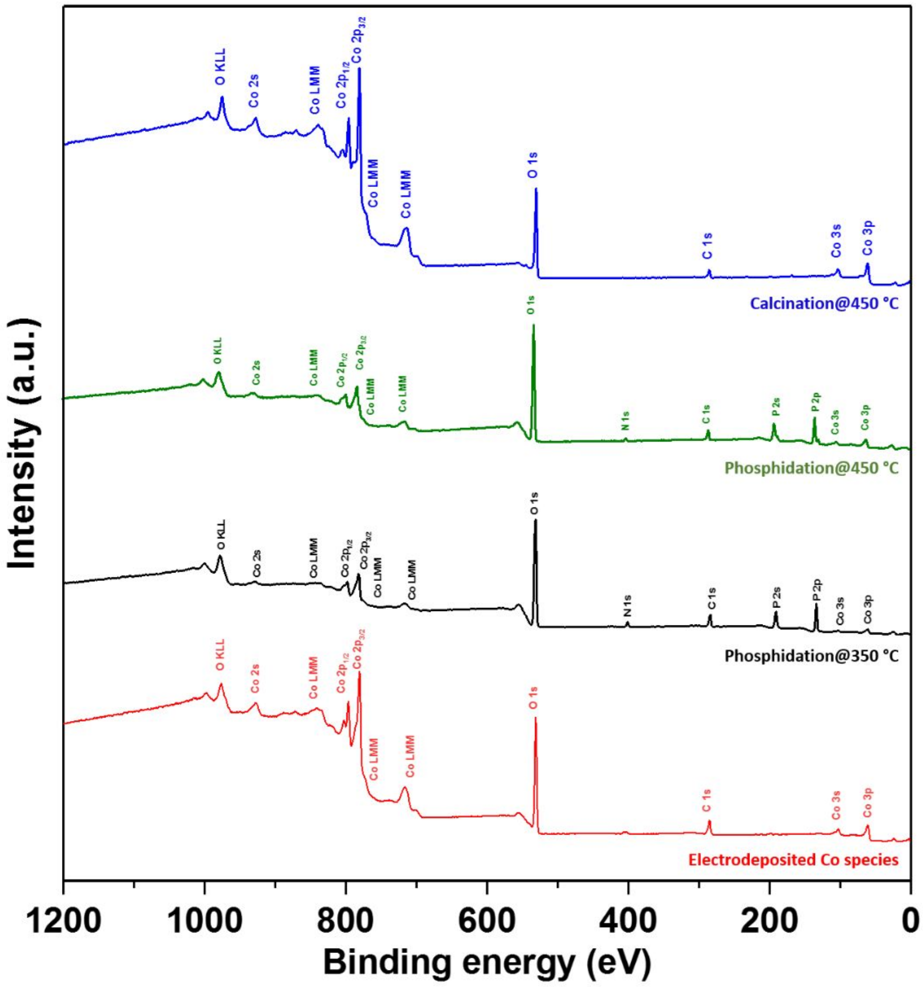

Figure S9. Survey spectrum of: Co-species/CC, phosphatized specimens at 350 °C and 450 °C, and calcined specimen at 450 °C.

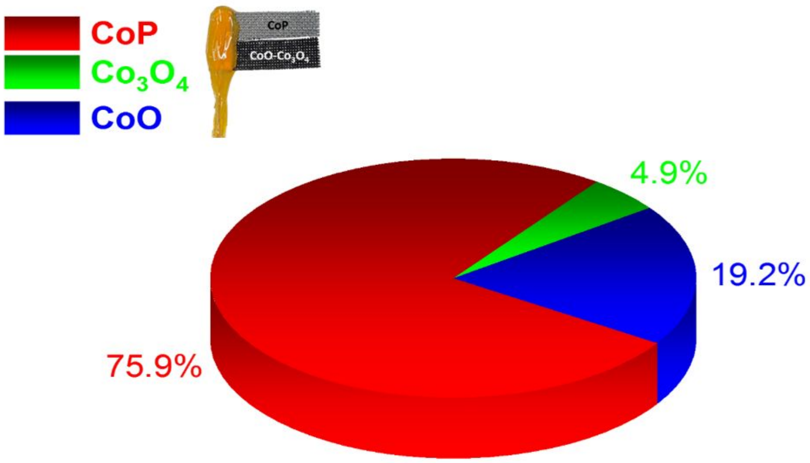

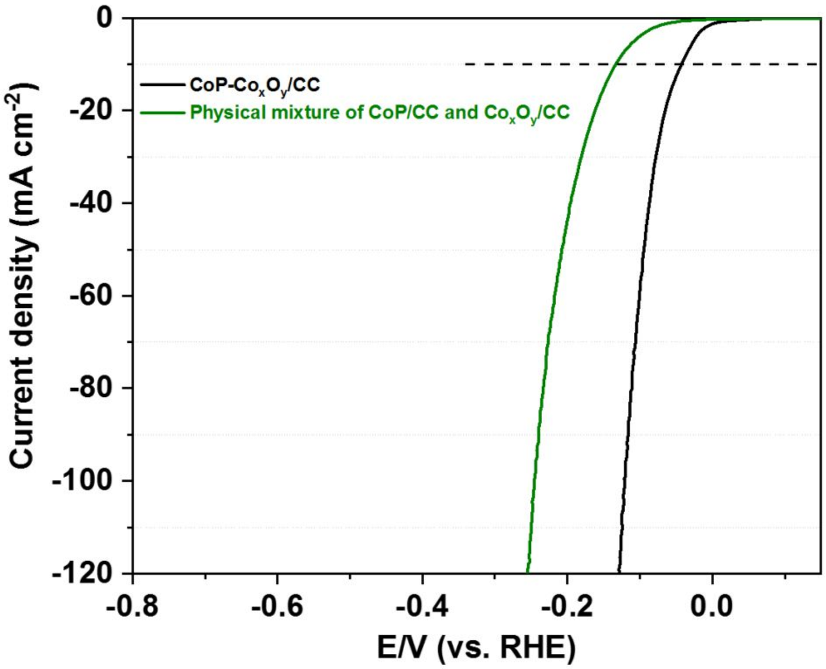

Figure S10. Polarization Curves at a scan rate of 0.1 mV s^{-1} in 1 M $KOH_{(aq)}$ electrolyte solution for CoP-Co_xO_y/CC (450 °C) in comparison with physical mixture of both CoP/CC and Co_xO_y/CC with corresponding mass ratio.

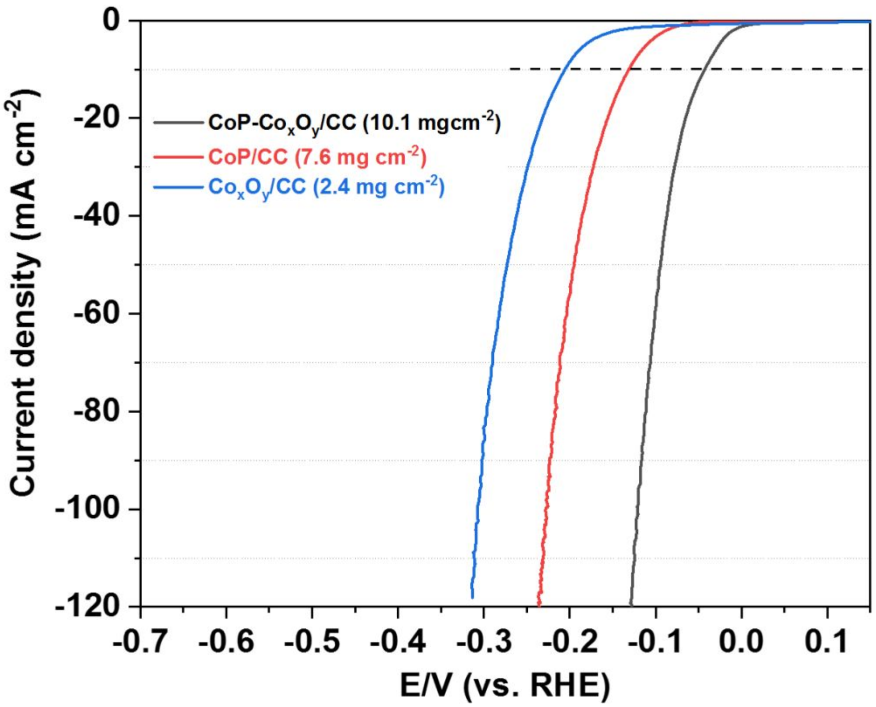

Figure S11. Polarization Curves at a scan rate of 0.1 mV s^{-1} in 1 M $KOH_{(aq)}$ electrolyte solution with corresponding mass loading: CoP-Co_xO_y/CC(450 °C), CoP/CC (850 °C), Co_xO_y/CC (450 °C).

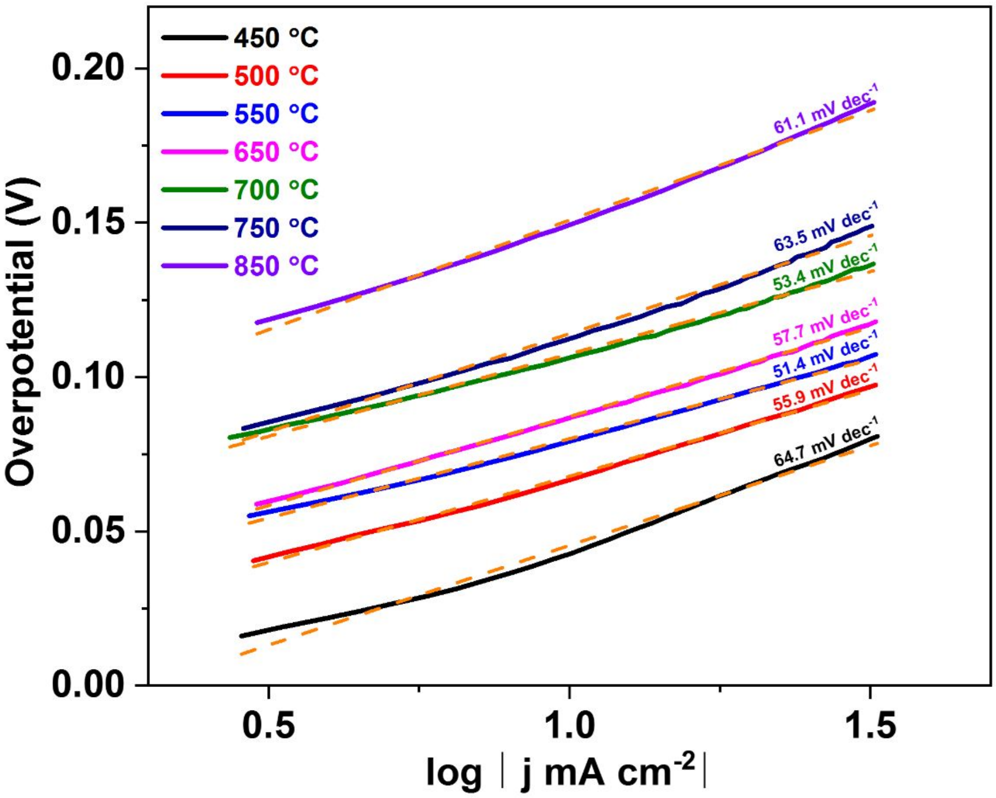

Figure S12. Tafel slopes extracted from the corresponding polarization curves.

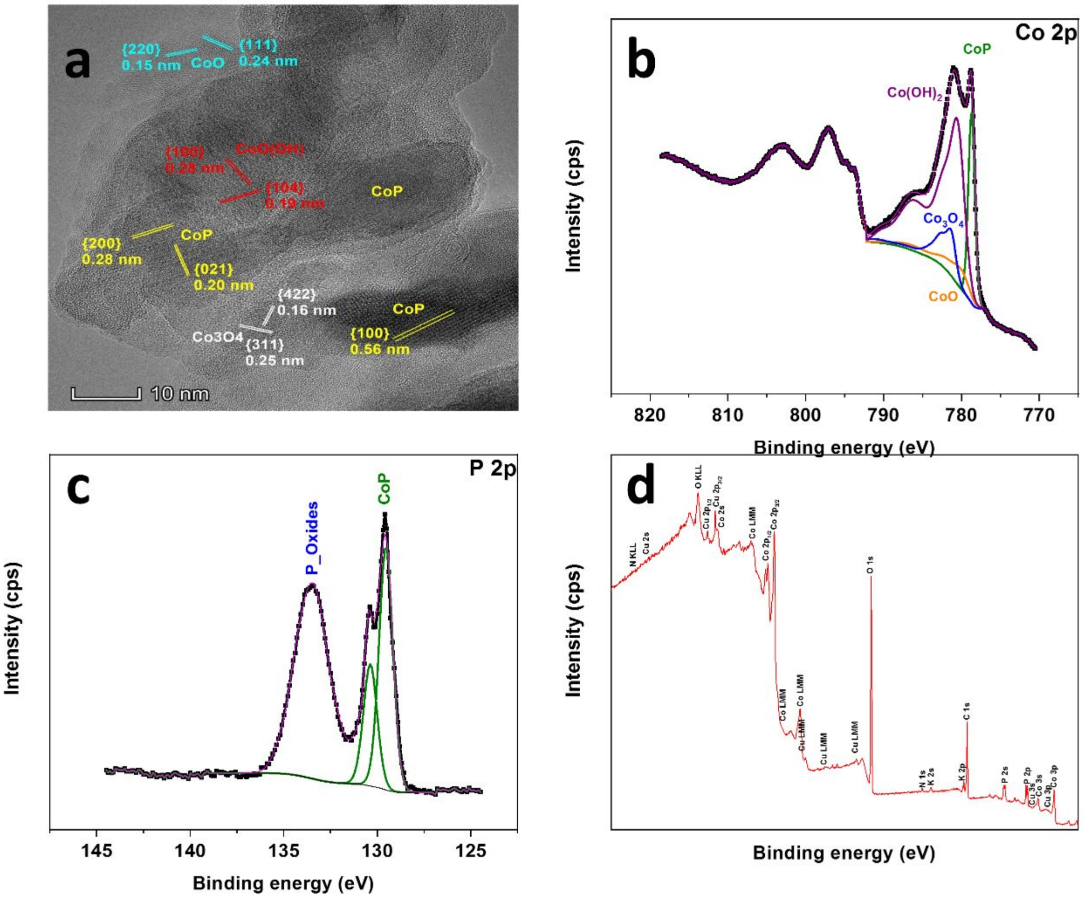

Figure S13. (a) HR-TEM image of post-catalytic specimen, CoP-Co_xO_y/CC, formed at 450 °C. (b and c) Narrow scan Co 2p and P 2p XPS spectra of post-catalytic specimen, CoP-Co_xO_y/CC, formed at 450 °C, respectively. (d) Survey spectrum of phosphatized specimen at 450 °C after being subjected to electrocatalytic analysis for 10 h.

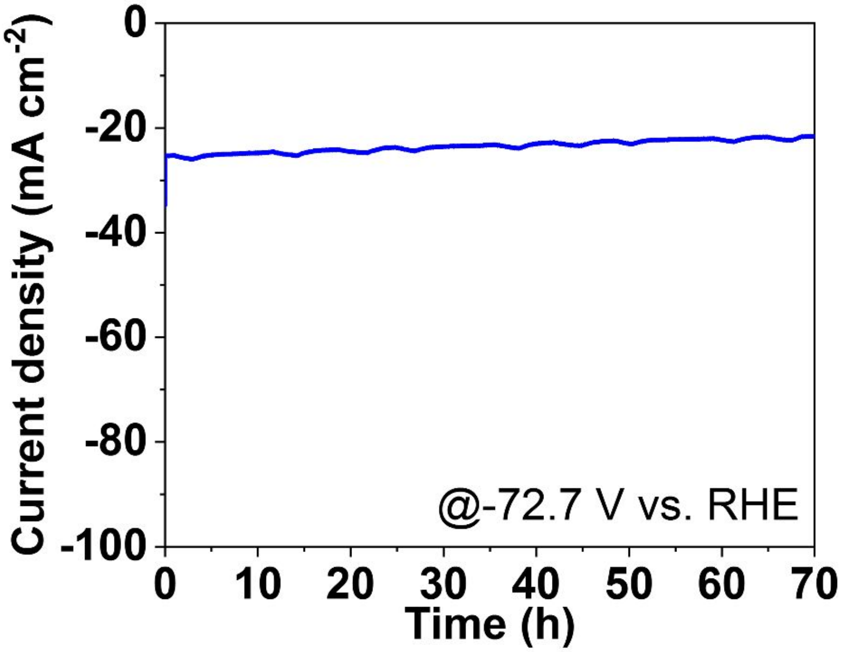

Figure S14. Time-dependent performance of CoP-Co_xO_y/CC (450 °C) in 1 M $KOH_{(aq)}$ solution for 70 hours at −72.7 mV (vs. RHE).

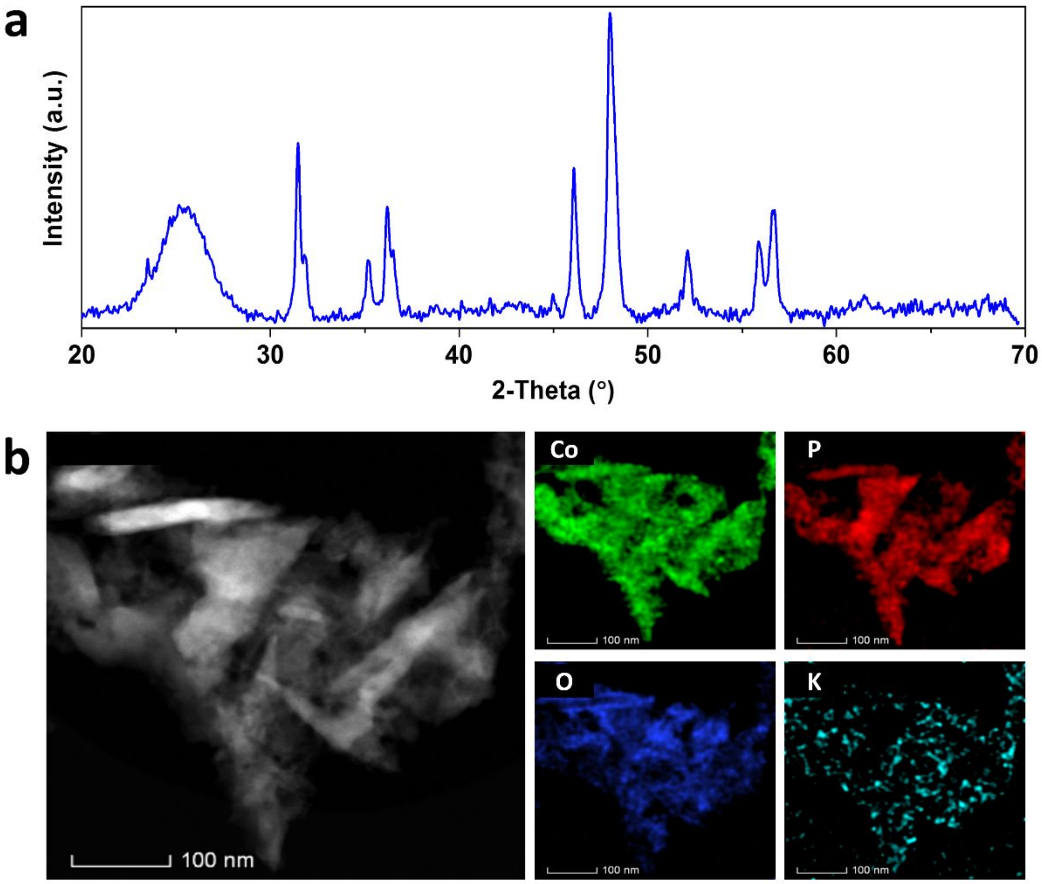

Figure S15. (a) XRD pattern of post-catalytic specimen, CoP-Co_xO_y/CC, formed at 450 °C. (b) EELS mapping of CoP-Co_xO_y/CC formed at 450 °C after being subjected to electrocatalytic analysis for 10 h.

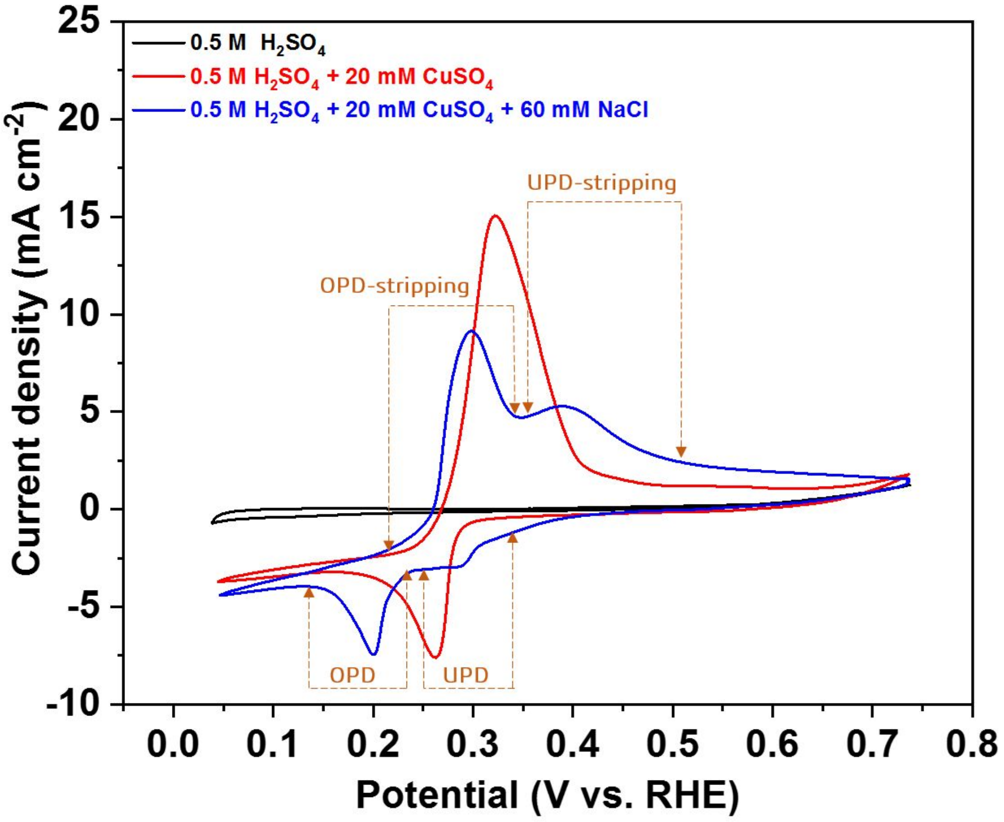

Figure S16. CV scan of 2 mg cm^{-2} Pt-C/CC electrocatalyst in different solutions.

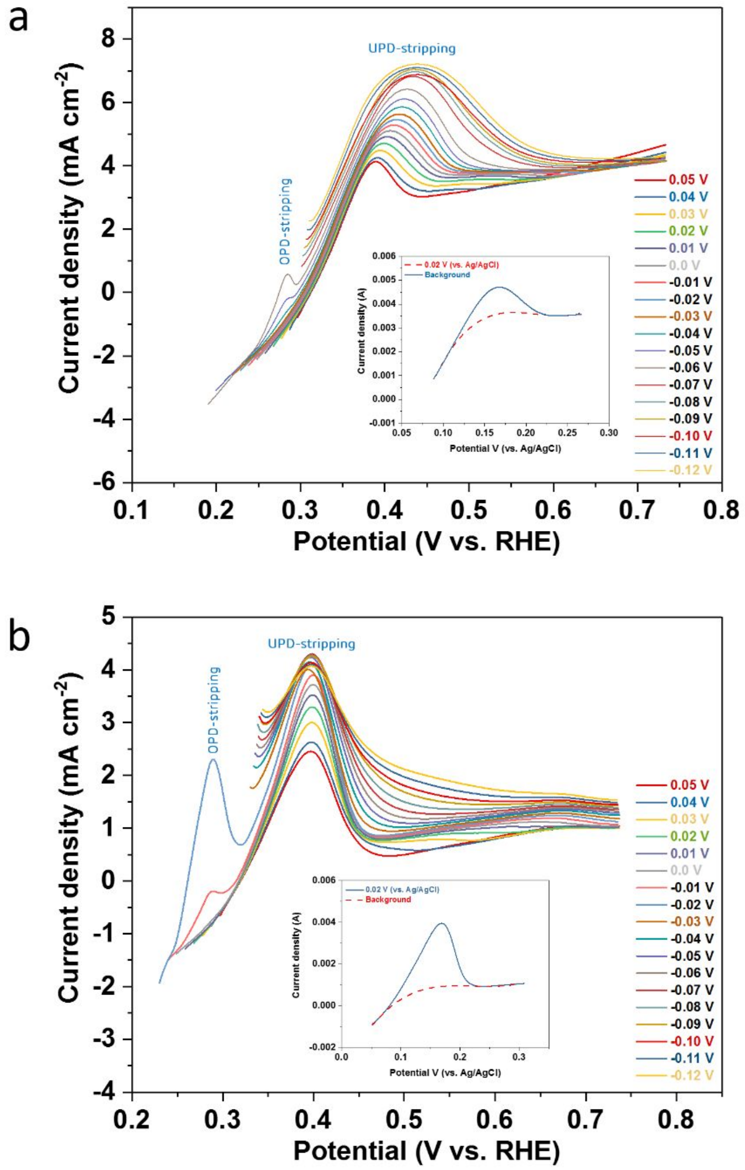

Figure S17. (a) LSV curves of stripping Cu deposited at CoP-CoxOy/CC electrocatalyst at different overpotentials (-0.12 V – 0.05 V (vs Ag/AgCl)) in 0.5 M H_2SO_4 + 20 mM $CuSO_4$ + 60 mM NaCl solution at scan rate of 2 mV s^{-1}. (b) LSV curves of stripping Cu deposited at 2 mg cm^{-2} Pt-C/CC electrocatalyst at different overpotentials (-0.12 V – 0.05 V (vs Ag/AgCl)) in 0.5 M H_2SO_4 + 20 mM $CuSO_4$ + 60 mM NaCl solution at scan rate of 2 mV s^{-1}. Inset in (a) and (b) shows typical selection for baseline of the LSV curve.

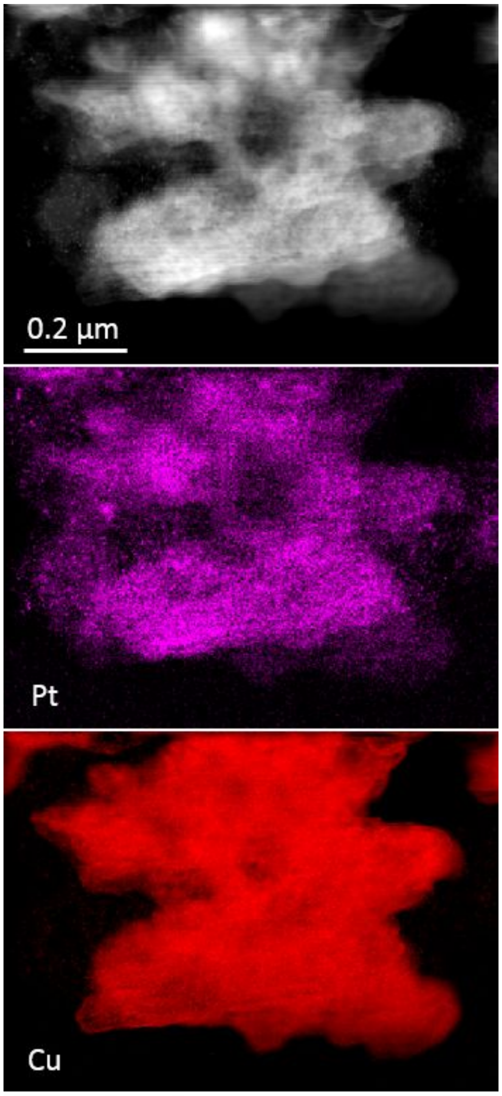

Figure S18. EELS mapping of Pt and UPD Cu of 2 mg cm^{-2} Pt-C/CC electrocatalyst.

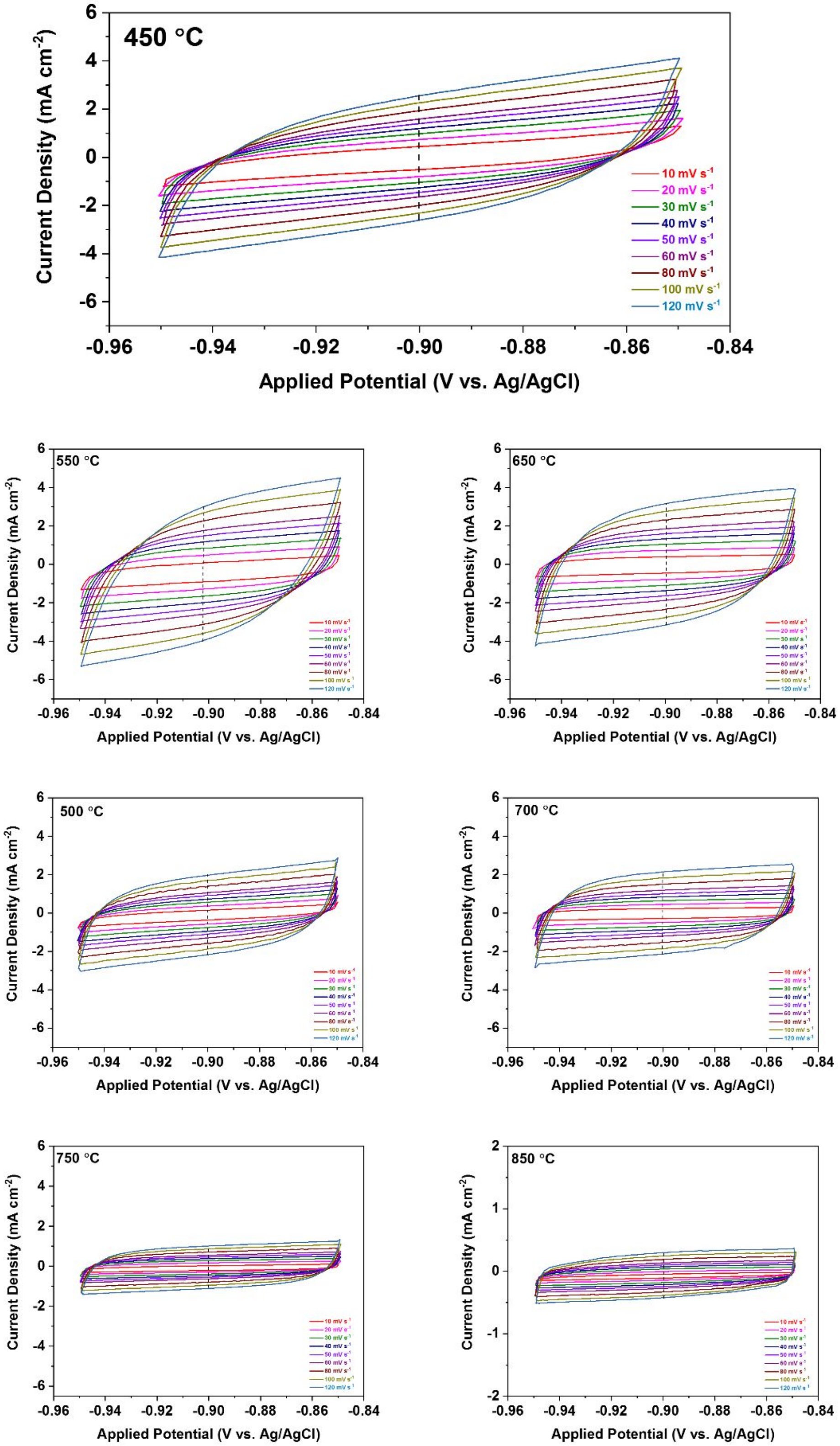

Figure S19. Cyclic voltammograms in the region of -0.95 and -0.85 V vs. Ag/AgCl of Co-P /CC at different phosphatization temperatures.

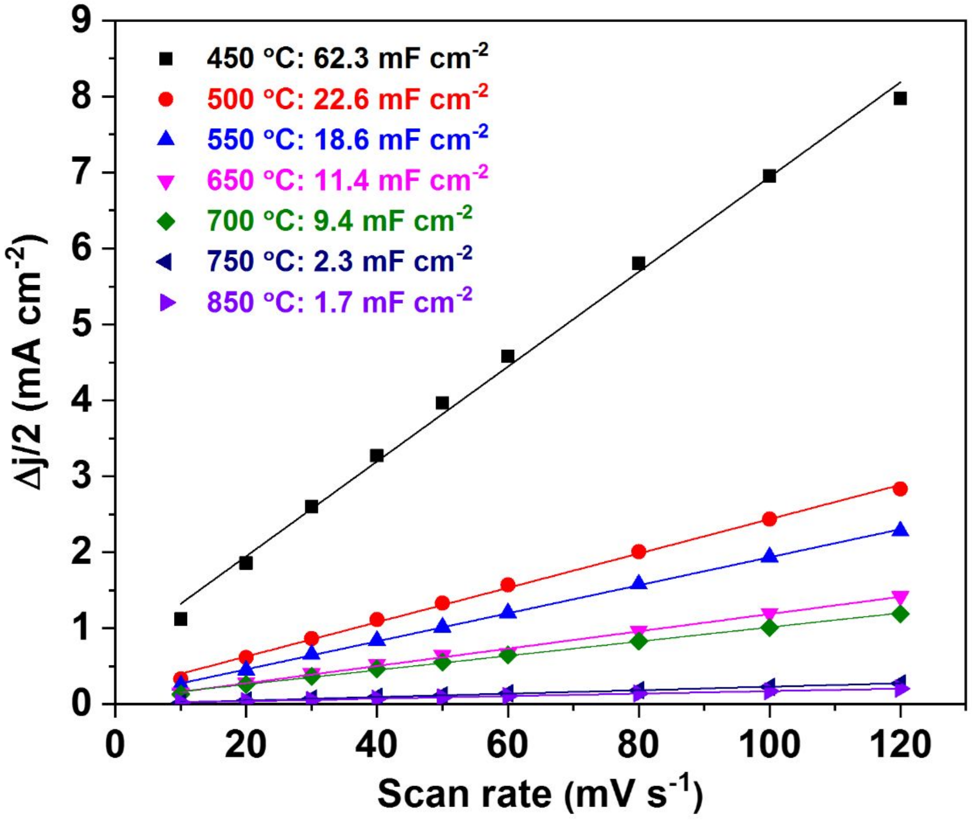

Figure S20. Double-layer capacitance (C_{dl}) of CoP_x/CC formed at different phosphatization temperatures.

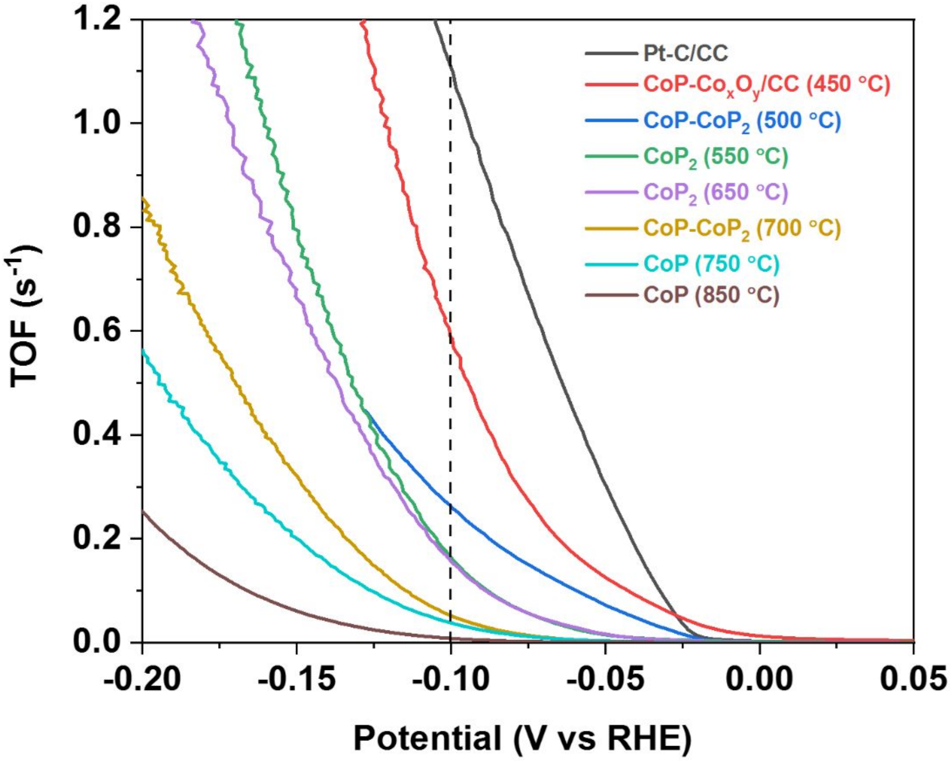

Figure S21. Turnover frequency (TOF) of Pt-C/CC and CoP_x/CC electrocatalysts formed at different phosphatization temperatures as a function of overpotential.

First-principles calculations

We analyzed the catalytic activities of the following heterostructures of CoP(110), CoO(110), and Co_3O_4(110) along with Co_3O_4-CoP, CoO-CoP, Co_3O_4-CoO-CoP and CoO-Co_3O_4-CoP **(Figure S22 (1-4))**. A good electrode material must possess a near-zero Gibbs free energy for the easy release of an H atom. The Gibbs free energy is defined as:

$$\Delta G_H^* = E_{ads} + \Delta ZPE - T\Delta S_H \quad (1)$$

where E_{ads} is the adsorption energy of a H atom, ΔZPE is the zero-point energy correction, T is the temperature in Kelvin, and ΔS_H is the entropy of a hydrogen molecule in the gas phase. The value of $\Delta ZPE - (300\ K) \times \Delta S_H$ can be approximated as 0.24 eV ($\Delta G_H^* = E_{ads} + 0.24$ eV). The adsorption energy is defined as:

$$E_{ads} = E_{heterostructure+H} - E_{heterostructure} - ½\, E_{H_2}, \quad (2)$$

where $E_{heterostructure+H}$ is the total energy of the relaxed heterostructure with the adsorbed H atom, $E_{heterostructure}$ is the total energy of the relaxed heterostructure, and $E_{H_2}$is the total energy of an isolated H_2 molecule in the gas phase. For all the heterostructures, we find that an H atom preferentially adsorbs on top of a Co atom. The obtained Gibbs free energies of a H atom adsorbed on the heterostructures are summarized in **Figure S22 b** and compared to pristine CoP. All heterostructures containing CoP (110) surface possess negative near-zero Gibbs free energy. The Gibbs free energy obtained for the CoO-CoP (-0.06 eV), CoO-Co_3O_4-CoP (-0.07 eV), and Co_3O_4-CoO-CoP (-0.08 eV) heterostructures are lower than the values reported for highly active HER electrocatalysts such as Pt (−0.09 eV), MoS_2 (0.08 eV), and WS_2 (0.22 eV).[6, 7] Further, we calculated the Bader charges [8] of the components of the heterostructures before and after H adsorption (see **Table S5**; normal and bold numbers are before and after H adsorption, respectively).

The results show that CoO and Co_3O_4 extracts charge from CoP. Additionally, we calculated the Bader charges of the surface atomic layers of the heterostructures, demonstrating more negative values for the CoP-Co_3O_4 and CoP-CoO-Co_3O_4 than those for the other heterostructures, which is the origin of the near-zero Gibbs free energy. Therefore, CoP enhances the catalytic activity for the HER.

Next, we determine the correlation between the binding energy of a water molecule and the Gibbs free energy (see **Figure S23** for the charge density differences induced by the adsorption of a water molecule on the heterostructures). The binding energy is defined as:

$$E_B = E_{heterostructure+H_2O} - E_{heterostructure} - E_{H_2O} \quad (3)$$

where $E_{heterostructure+H_2O}$ is the total energy of a relaxed heterostructure with the adsorbed water molecule, $E_{heterostructure}$ is the total energy of the relaxed heterostructure, and E_{H_2O} is the total energy of an isolated water molecule. We find that a low binding energy is not compatible with a near-zero Gibbs free energy (see **Table S5**) but results in low catalytic activity for the HER.

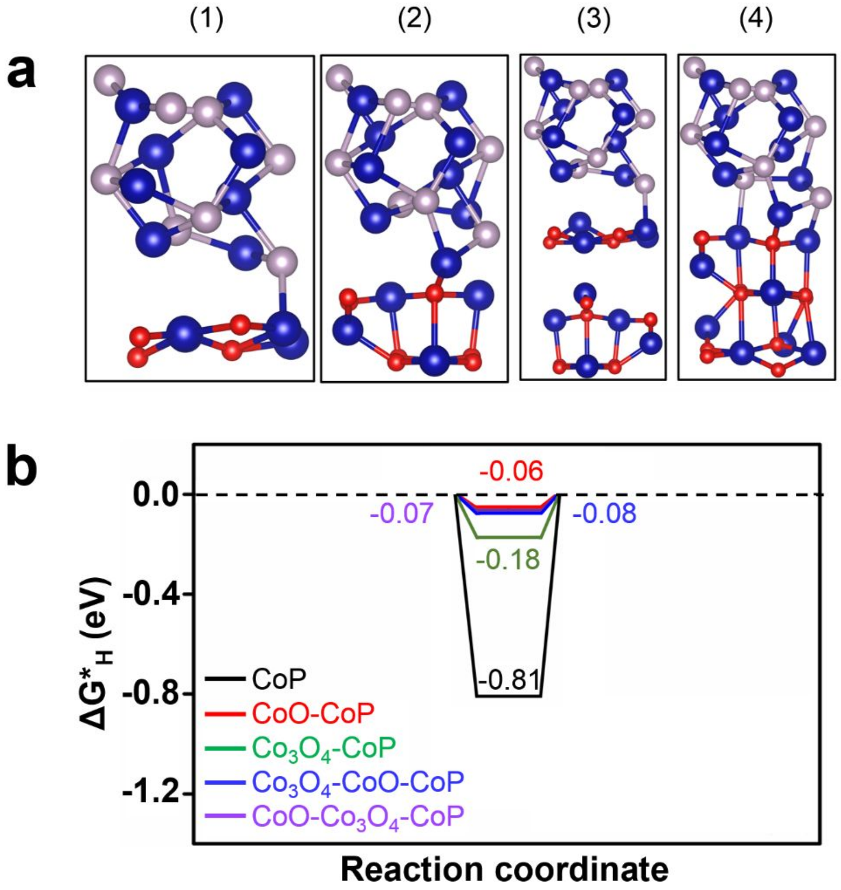

Figure S22. (a) Heterostructure models of (1) CoO-CoP, (2) Co_3O_4-CoP, (3) Co_3O_4-CoO-CoP, and (4) CoO-Co_3O_4-CoP. Blue, purple, and red spheres represent Co, P, and O atoms, respectively. (b) Gibbs free energy of a H atom adsorbed on isolated CoP and the CoO-CoP, Co_3O_4-CoP, Co_3O_4-CoO-CoP and CoO-Co_3O_4-CoP heterostructures.

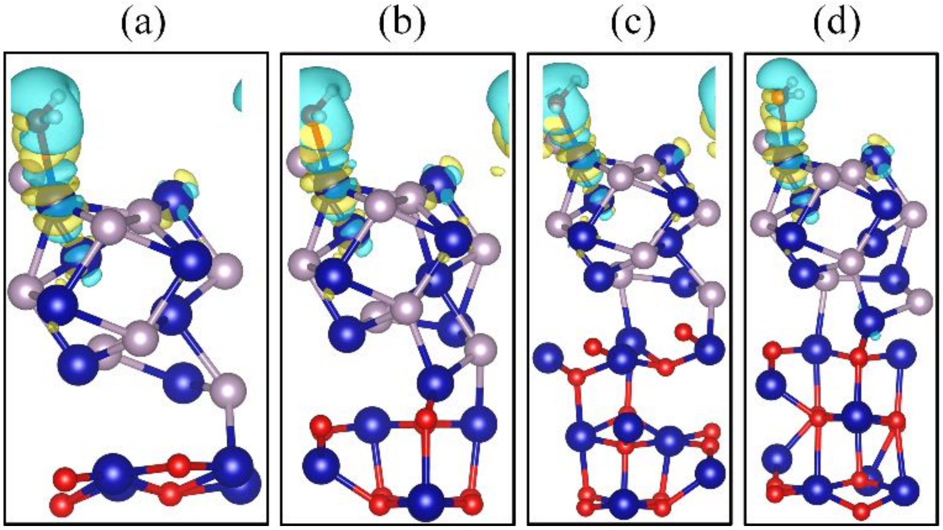

Figure S23. Charge density differences induced by the adsorption of a water molecule on the (a) CoO-CoP, (b) Co_3O_4-CoP, (c) Co_3O_4-CoO-CoP and (d) CoO-Co_3O_4-CoP heterostructures. Accumulation and depletion are denoted as yellow and blue isosurfaces (isovalue 3×10^{-3} electrons bohr^{-3}).

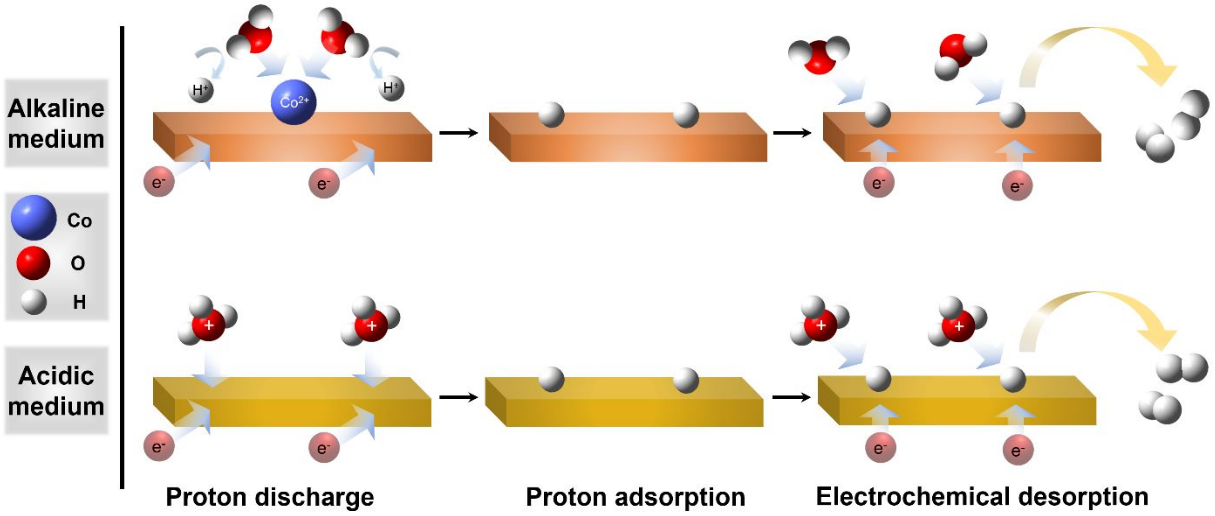

Scheme 1. Volmer-Heyrovsky mechanism of the HER on the surface of a catalyst in basic solution.

Table S1. Detailed characterization of $Co(OH)_2$, Co_xO_y, and CoP_x nanoparticles according to XRD analysis.

Phosphatization temperatures (°C)	The compounds		JCPDS card no.
	Name	Chemical formula	
23	Cobalt hydroxide	$Co(OH)_2$	01-072-1474
450	Cobalt oxide	CoO	00-043-1004
	Tri-cobalt tetra-oxide	Co_3O_4	01-071-0816
	Cobalt phosphide	CoP	03-065-1474
550, 650	Cobalt di-phosphide	CoP_2	01-077-0263
750, 850	Cobalt phosphide	CoP	01-089-4862
500, 700	Cobalt phosphide	CoP	03-065-2593
	Cobalt di-phosphide	CoP_2	01-077-0263

Table S2. Detailed characterization of CoP-Co_xO_y, CoP_2, and CoP formed at different phosphatization temperatures according to XRD analysis.

$CoP-Co_3O_4-CoO$				CoP_2				CoP			
2θ	h	k	l	2θ	h	k	l	2θ	h	k	l
23.660	1	0	1	24.805	-1	1	1	48.119	2	1	1
46.239	1	1	2	32.238	0	2	0	23.660	1	0	1
48.119	2	1	1	35.165	0	0	2	31.598	0	1	1
52.280	1	0	3	35.578	2	0	0	32.013	0	0	2
56.010	0	2	0	37.616	-1	2	1	35.329	2	0	0
56.798	3	0	1	38.835	0	1	2	36.322	1	1	1
31.346	2	2	0	39.214	2	1	0	36.689	1	0	2
36.936	3	1	1	41.704	-2	1	2	45.122	2	1	0
38.642	2	2	2	46.079	1	0	2	46.239	1	1	2
55.797	4	2	2	48.764	2	2	0	52.280	1	0	3
59.510	5	1	1	48.446	0	2	2	56.010	0	2	0
65.408	4	4	0	51.692	-1	1	3	56.798	0	1	3
42.402	2	0	0	50.878	-2	2	2				
61.521	2	2	0	52.298	-3	1	1				
				53.172	-1	3	1				
				58.516	1	3	1				
				61.543	2	0	2				
				61.925	0	3	2				

Table S3. Summary of HER performance for recent reports Co-based electrocatalysts.

Catalyst	Substrate	η_{10} (mV)	Tafel slope (mV/dec)	Electrolyte	Reference
$CoP\text{-}Co_xO_y$	CC	-43	43.0	1 M $KOH_{(aq)}$	This work
f-CoP/CoP_2	Al_2O_3	-138	73	1 M $KOH_{(aq)}$	[9]
Co_2P	CP	-70	59.7	1 M $KOH_{(aq)}$	[10]
Co_2P	CP	-120	48	0.5 M $H_2SO_{4(aq)}$	[10]
CoP_2/RGO	GCE	-88	50	1 M $KOH_{(aq)}$	[11]
CoP	CC	-48	42.6	1 M $KOH_{(aq)}$	[12]
Co-P	Cu foil	-94	42	1 M $KOH_{(aq)}$	[13]
Co_3O_4/MoS_2	Ni foam	-205	128	1 M $KOH_{(aq)}$	[14]
CoP_3	CFP	-124	88	1 M $KOH_{(aq)}$	[15]
CoP_3	CFP	-78	53	0.5 M $H_2SO_{4(aq)}$	[15]
$Co(OH)_x$/CoP	GCE	-100	76	1 M $KOH_{(aq)}$	[16]
Ni/NiCoP	Ti plate	-90	95	1 M $KOH_{(aq)}$	[17]
Co_2P/Co_3O_4	Ti plate	-86	73	1 M $KOH_{(aq)}$	[18]

CC: carbon cloth; CP: carbon paper; GCE: glassy carbon electrode; CFP: carbon fiber paper

Table S4. Loading amount of CoP_x/CC formed during phosphatization at various temperatures.

Mass of catalyst (mg)	CoP-Co_xO_y 450°C	CoP/CoP_2 500°C	CoP_2 550°C	CoP_2 650 °C	CoP 700 °C	CoP 750 °C	CoP 850 °C
Pristine CC	31.3	30.7	31.5	30.3	30.5	31.6	31.9
Before phosphatization	47.5	46.9	45.5	46.6	47.7	46.2	47.0
After phosphatization	51.5	50.3	51.7	49.5	49.4	51.5	51.8
Loading amount(mg/cm^2)	10.1	9.8	10.1	9.6	9.45	9.95	9.95

Table S5. Gibb's free energy of a H atom adsorbed on the heterostructure, Bader charges of the components and the surface atomic layer of the heterostructure (normal and bold numbers are before and after H adsorption, respectively), and binding energy of H_2O on the heterostructure.

Heterostructure	Gibbs Free Energy	Bader Charge (electrons)				Binding energy (eV)
		CoP	CoO	Co_3O_4	Surface atomic layer	
CoO-CoP	–0.06	+0.10/**+0.21**	–0.10/**–0.11**	--/--	–0.17/**–0.05**	–1.94
Co_3O_4-CoP	–0.18	+0.15/**+0.49**	--/--	–0.15/**–0.15**	–0.19/**–0.11**	–0.86
Co_3O_4-CoO-CoP	–0.08	+0.03/**+0.83**	–0.10/**+0.44**	+0.07/**–0.93**	–0.15/**+0.04**	–2.33
CoO-Co_3O_4-CoP	–0.07	+0.48/**+0.83**	+0.84/**+0.84**	–1.32/**–1.34**	–0.18/**+0.22**	–0.65

References:

1. Kresse, G.; Joubert, D. From Ultrasoft Pseudopotentials to the Projector Augmented-Wave Method. *Phys. Rev. B* **1999**, *59*(3), 1758-1775.
2. Blochl, P. E. Projector Augmented-Wave Method. *Phys. Rev. B* **1994**, *50*(24), 17953-17979.
3. Grimme, S.; Antony, J.; Ehrlich, S.; Krieg, H. A Consistent and Accurate ab initio Parametrization of Density Functional Dispersion Correction (DFT-D) for the 94 elements H-Pu. *J. of Chem. Phys.* **2010**, *132*(15), 154104-154123.
4. Hu, G. X.; Tang, Q.; Jiang, D. E. CoP for Hydrogen Evolution: Implications from Hydrogen Adsorption. *Phys. Chem. Chem. Phys.* **2016**, *18*(34), 23864-23871.
5. Jin, X.; Huai, L. Y.; Wen, H.; Yi, W. C.; Liu, J. Y. Reduction of NO with CO on the Co_3O_4(110)-B and CoO(110) Surfaces: A First-Principles Study. *J. Phys. Chem. C* **2019**, *123*(3), 1770-1778.
6. Bonde, J.; Moses, P. G.; Jaramillo, T. F.; Norskov, J. K.; Chorkendorff, I. Hydrogen Evolution on Nano-Particulate Transition Metal Sulfides. *Faraday Discuss.* **2008**, *140*, 219-231.
7. Hinnemann, B.; Moses, P.; Bonde, J.; Jørgensen, K.; Nielsen, J-H.; Horch, S.; Chorkendorff, I.; Nørskov, J. K. Biomimetic Hydrogen Evolution: MoS_2 Nanoparticles as Catalyst for Hydrogen evolution. *J. Am. Chem. Soc.* **2005**, *127*(15), 5308-5309.
8. Tang, W.; Sanville, E.; Henkelman, G. A. Grid-Based Bader Analysis Algorithm without Lattice Bias. *J. Phys.: Condens. Matter* **2009**, *21*(8), 084204-084211.
9. Li, W.; Zhang, S. L.; Fan, Q. N.; Zhang, F. Z.; Xu, S. L. Hierarchically Scaffolded CoP/CoP_2 nanoparticles: Controllable Synthesis and their Application as a Well-Matched Bifunctional Electrocatalyst for Overall Water Splitting. *Nanoscale* **2017**, *9*(17), 5677-5685.
10. Zhang, Y.; Gao, L.; Hensen, E. J. M.; Hofmann, J. P. Evaluating the Stability of Co_2P Electrocatalysts in the Hydrogen Evolution Reaction for Both Acidic and Alkaline Electrolytes. *ACS Energy Lett.* **2018**, *3*(6),1360-1365.
11. Wang, J. M.; Yang, W. R.; Liu, J. Q. CoP_2 Nanoparticles on Reduced Graphene Oxide Sheets as a Super-Efficient Bifunctional Electrocatalyst for Full Water Splitting. *J. Mater. Chem. A* **2016**, *4*(13), 4686-4690.
12. Yang, X. L.; Lu, A. Y.; Zhu, Y. H.; Hedhili, M. N.; Min, S. X.; Huang, K. W.; Han, Y.; Lin, L. J. CoP Nanosheet Assembly Grown on Carbon Cloth: A Highly Efficient Electrocatalyst for Hydrogen Generation. *Nano Energy* **2015**, *15*, 634-641.
13. Jiang, N.; You, B.; Sheng, M. L.; Sun, Y. J. Electrodeposited Cobalt-Phosphorous-Derived Films as Competent Bifunctional Catalysts for Overall Water Splitting. *Angew. Chem. Int. Ed.* **2015**, *54*(21), 6251-6254.
14. Muthurasu, A.; Maruthapandian, V.; Kim, H. Y. Metal-Organic Framework Derived Co_3O_4/MoS_2 Heterostructure for Efficient Bifunctional Electrocatalysts for Oxygen Evolution Reaction and Hydrogen Evolution Reaction. *Appl. Catal. B* **2019**, *248*, 202-210.
15. Wu, T. L.; Pi, M. Y.; Wang, X. D.; Zhang, D. K.; Chen, S. J. Three-Dimensional Metal-Organic Framework Derived Porous CoP_3 Concave Polyhedrons as Superior Bifunctional Electrocatalysts for the Evolution of Hydrogen and Oxygen. *Phys.Chem. Chem. Phys.* **2017**, *19*(3), 2104-2110.

16. Su, L.; Cui, X. Z.; He, T.; Zeng, L. M.; Tian, H.; Song, Y. L.; Qi, K.; Xia, B. Y. Surface Reconstruction of Cobalt Phosphide Nanosheets by Electrochemical Activation for Enhanced Hydrogen Evolution in Alkaline Solution. *Chem. Sci.* **2019**, *10*(7), 2019-2024.
17. Lin, Y.; Pan, Y.; Liu, S. J.; Sun, K. A.; Cheng, Y. S.; Liu, M.; Wang, Z. J.; Li, X. Y.; Zhang, J. Construction of Multi-Dimensional Core/Shell Ni/NiCoP Nano-Heterojunction for Efficient Electrocatalytic Water Splitting. *Appl. Catal. B* **2019**, *259*, 118039- 118047.
18. Yu, X. T.; Wang, M. Y.; Gong, X. Z.; Guo, Z. C.; Wang, Z.; Jiao S. Q. Self-Supporting Porous CoP-Based Films with Phase-Separation Structure for Ultrastable Overall Water Electrolysis at Large Current Density. *Adv. Energy Mater.* **2018**, *8*(34), 1802445-1802455.
